# Supplementary material for: Maynard Smith revisited: A multi-agent reinforcement learning approach to the coevolution of signalling behaviour
Source: PLoS Comput Biol. 2025 Aug 26;21(8):e1013302. doi: 10.1371/journal.pcbi.1013302 (PMC12440204; doi:10.1371/journal.pcbi.1013302)
Supplement: S1 Appendix — (PDF) [file pcbi.1013302.s001.pdf]

S1 Appendix: Derivation of evolutionarily stable strategies

In the original paper presenting the Sir Philip Sidney game [1], Maynard Smith derives the evolutionarily stable strategy for each of the players in the game, and the threshold values that maintain this equilibrium. As long as the thresholds are met, the evolutionarily stable strategy for the beneficiary is to signal only when thirsty, whereas for the donor it is to give only if the beneficiary signals.

These thresholds can be summarised as follows. It is evolutionarily stable for B to only signal when thirsty if:

$$1 - c + rU > r \tag{1a}$$

$$V + r > 1 - c + rU \tag{1b}$$

It is evolutionarily stable for D to give only when B signals if:

$$1 + rV > U + r \tag{2a}$$

$$U + r(1 - c) > 1 \tag{2b}$$

When these inequalities are satisfied, Maynard Smith claimed that the resulting equilibrium supports Zahavi's handicap principle, and ensures that honest signalling is observed within the given constraints. If one or more of these inequalities do not hold, other strategies will invade - i.e. it may be more evolutionarily stable for the beneficiary to always signal (leading to deceptive signalling), or for the donor to never give the resource. These results have previously been shown both theoretically [2] and through simulation [3], and are also supported by later analyses of the Philip Sidney game, including continuous variants of the model [4, 5]. These models assume infinite populations; it is worth noting that a reinforcement learning approach to the problem found much lower prevalence of honest signalling within finite populations [6].

However, to arrive at the evolutionarily stable strategy for each of the players, it is assumed that the other is already playing their evolutionarily stable strategy. In other words, to derive when it would be evolutionarily stable for B to signal only when thirsty, it is assumed that D will only give if B signals, and vice versa. This formulation therefore overlooks some possible outcomes, namely those where the opponent is not following the predefined strategy. To illustrate this, we can first consider the edge cases where $p = 1$ or $p = 0$; Maynard Smith states in the original derivation that the value of p does not affect the results. We therefore have two possible matrices for inclusive fitness, one when B is always thirsty, and one when B is never thirsty. These are visualised in Tables 1 and 2.

Table 1: Matrix of inclusive fitness given $p = 1$ (B is thirsty with probability 1).

		D	
		Keep	*Give*
B	*Signal*	r, 1	$1 - c + rU$, $U + r(1 - c)$
	Not signal	r, 1	$1 + rU$, $U + r$

Taking the first case of $p = 1$, where B is thirsty (Table 1), we can consider each player's actions. B plays first and can choose whether to signal or not. Given the matrix of inclusive fitness, B is indifferent in the case that D chooses to keep the resource (the resulting fitness will be r either way). Looking at the second column showing the inclusive fitness if D does give

Table 2: Matrix of inclusive fitness given $p = 0$ (B is not thirsty with probability 1).

		D	
		Keep	*Give*
B	*Signal*	$r + V(1-c)$, $1 + rV(1-c)$	$1 - c + rU$, $U + r(1-c)$
	Not signal	$r + V$, $1 + rV$	$1 + rU$, $U + r$

the resource, we can see B would always prefer not to have signalled. The possible payoffs are $1 - c + rU$ or $1 + rU$. As we have established that $0 < c < 1$, the inclusive fitness had B not signalled will always be greater. The same can be shown in the case that $p = 0$ (Table 2). The matrix of inclusive fitness illustrates that whatever action D takes, B's inclusive fitness is always higher if they have chosen not to signal.

Given that B would always choose not to signal, we can then consider D's preference. In the case of $p = 1$, the possible payoffs are 1 if they keep the resource, and $U + r$ if they give; therefore, if $U + r > 1$, D would always choose to give the resource, even though B did not signal. In the case of $p = 1$, we require $U + r > 1 + rV$ to get the same result.

Going back to the general case, where the value of p is not assumed, we can then derive the threshold values of the different parameters for this to be the evolutionarily stable set of strategies. B knows the state they are in, therefore the relevant inequalities are as follows. B would always choose not to signal as long as:

$$1 + rU > 1 - c + rU \tag{3a}$$

$$r + V > r + V(1-c) \tag{3b}$$

which can be simplified to:

$$c > 0 \text{ and } V > 0 \tag{3c}$$

Both of these conditions will always be satisfied, as we have established that the signal must be costly (so we always have $c > 0$), and that $0 < V < 1$. The inequalities are also satisfied if $c < 0$ and $V < 0$, however these are not possible values for the parameters. Therefore, in all possible combinations U, V, c and r, the evolutionarily stable strategy for B is not to signal. When it comes to D, the value of p also has to be taken into account as they do not know B's state. Given that B signalled, the stable strategy for D would be to give the resource as long as we have:

$$p(U + r(1-c)) + (1-p)(U + r(1-c)) > p(1) + (1-p)(1 + rV(1-c)) \tag{4a}$$

Which can be simplified to:

$$U + r(1-c) > 1 + rV(1-c)(1-p) \tag{4b}$$

Conversely, given that B did not signal, the stable strategy for D would be to keep the resource as long as we have:

$$p(U + r) + (1-p)(U + r) > p(1) + (1-p)(1 + rV) \tag{5a}$$

Which can be simplified to:

$$r > \frac{1 - U}{1 - V + pV} \tag{5b}$$

As has been shown above, the evolutionarily stable strategy for B is never to signal. Assuming this, the only condition that must be met is Eq (5b). An example set of values that satisfy the inequality are: $r = 0.8$, $p = 0.5$, $U = 0.9$, $V = 0.1$; this holds for varying signal costs, including $c = 0.25$ and $c = 0.75$.

References

[1] John Maynard Smith. “Honest signalling: the Philip Sidney game”. In: *Animal Behaviour* 42 (1991), pp. 1034–1035.

[2] Carl T. Bergstrom and Michael Lachmann. “Signalling among relatives. I. Is costly signalling *too* costly?” In: *Philosophical Transactions of the Royal Society of London. Series B: Biological Sciences* 352.1353 (1997), pp. 609–617.

[3] Steven Hamblin and Peter L. Hurd. “When will evolution lead to deceptive signaling in the Sir Philip Sidney game?” In: *Theoretical Population Biology* 75.2 (2009), pp. 176–182.

[4] Rufus A. Johnstone and Alan Grafen. “The continuous Sir Philip Sidney game: A simple model of biological signalling”. In: *Journal of Theoretical Biology* 156.2 (1992), pp. 215–234.

[5] John Maynard Smith and David Harper. *Animal Signals.* Oxford: Oxford University Press, 2003.

[6] David Catteeuw, Bernard Manderick, and The Anh Han. “Evolutionary stability of honest signaling in finite populations”. In: *2013 IEEE Congress on Evolutionary Computation.* 2013, pp. 2864–2870.
