# Supplementary material for: Maynard Smith revisited: A multi-agent reinforcement learning approach to the coevolution of signalling behaviour
Source: PLoS Comput Biol. 2025 Aug 26;21(8):e1013302. doi: 10.1371/journal.pcbi.1013302 (PMC12440204; doi:10.1371/journal.pcbi.1013302)
Supplement: S2 Appendix — (PDF) [file pcbi.1013302.s002.pdf]

S2 Appendix: Results with varying learning and discount rates

Results from varying the learning and discount rates in Case 1: $U = 0.2, V = 0.2, r = 0.5$.

1 Learning rate = 0.1

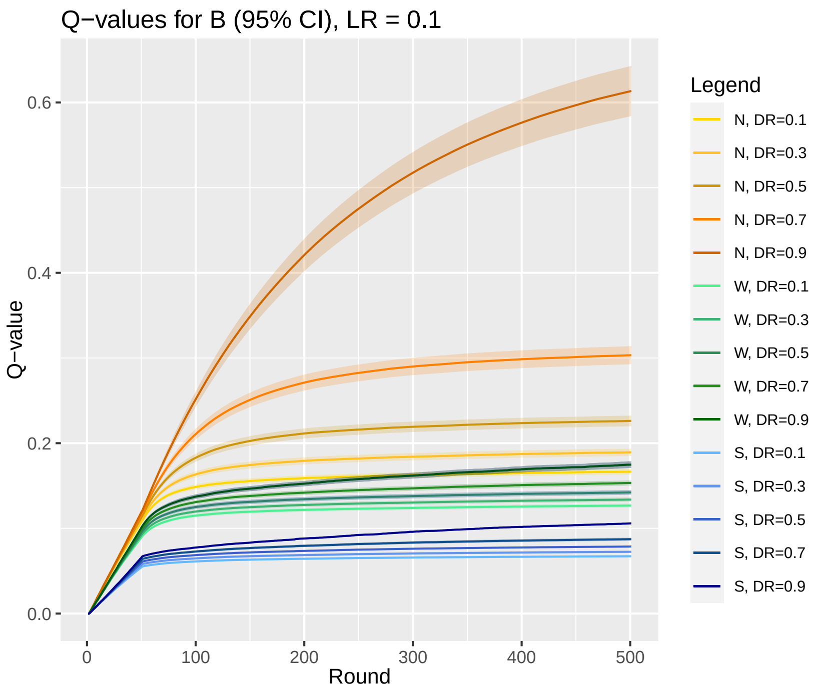

(a) Player B

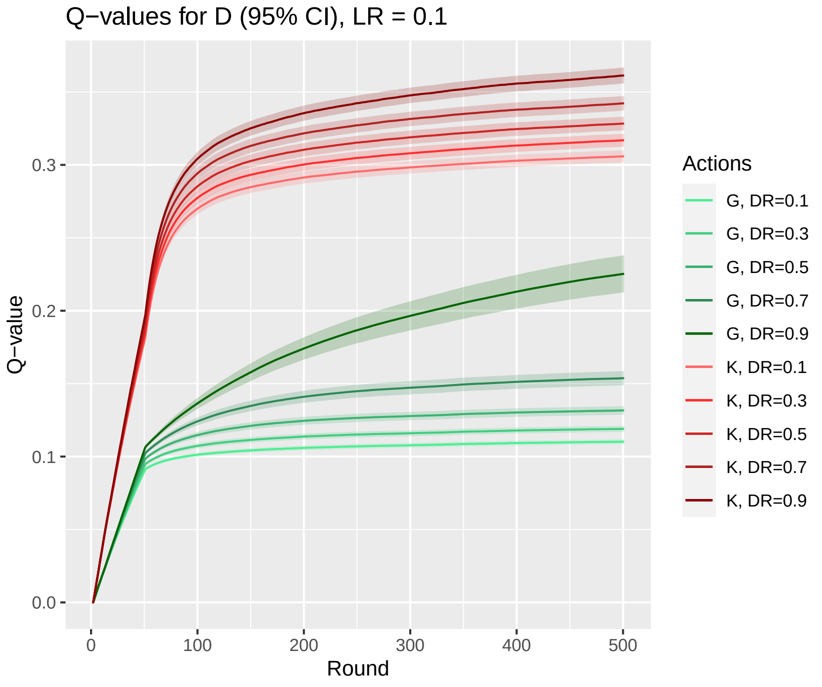

(b) Player D

Figure 1: Case 1 resulting strategies with learning rate of 0.1 and varying discount rates.

2 Learning rate = 0.3

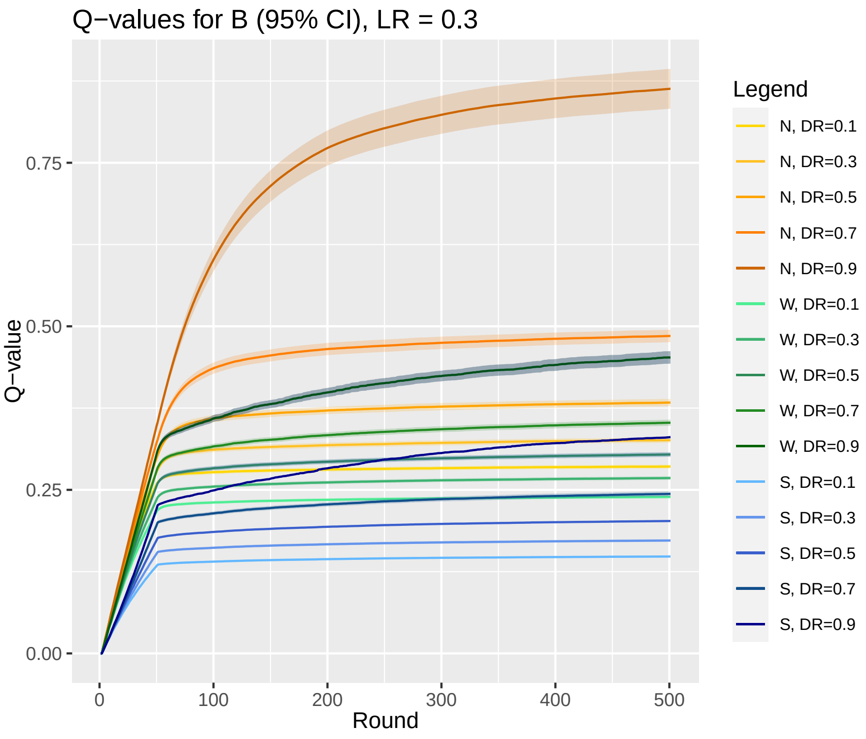

(a) Player B

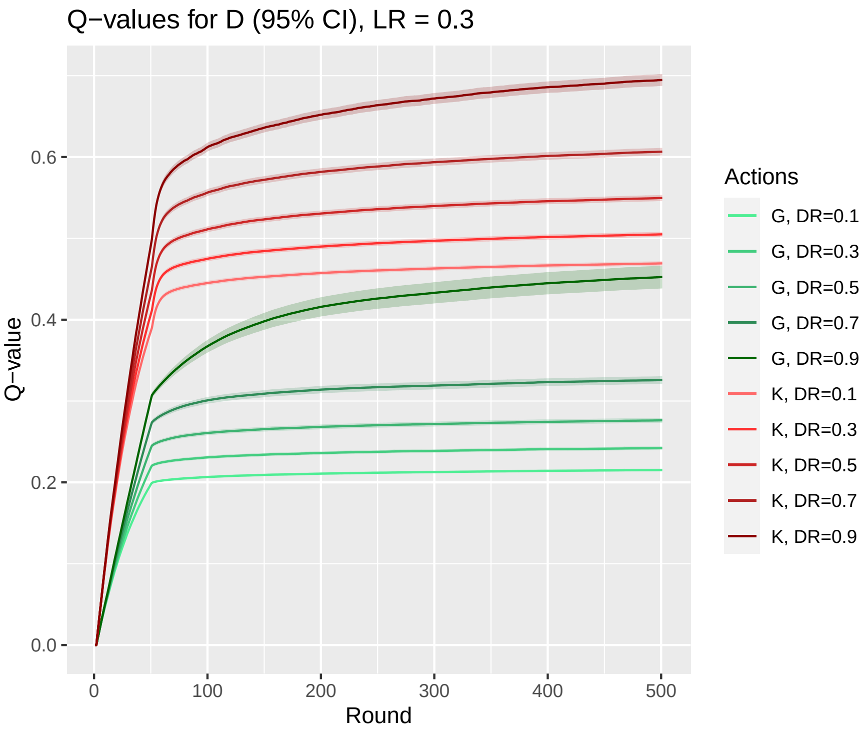

(b) Player D

Figure 2: Case 1 resulting strategies with learning rate of 0.3 and varying discount rates.

3 Learning rate = 0.5

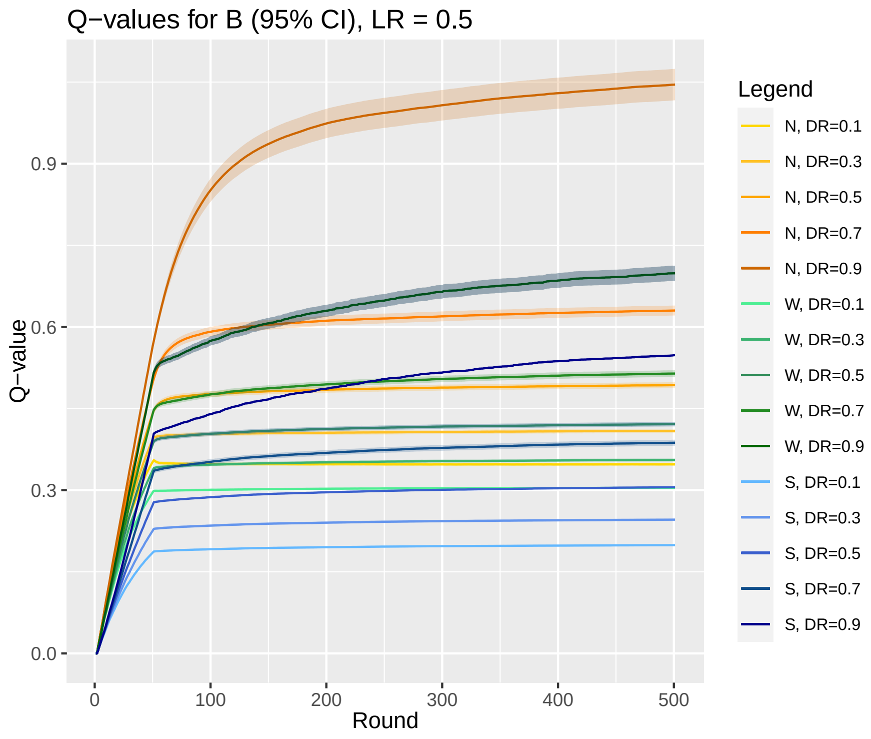

(a) Player B

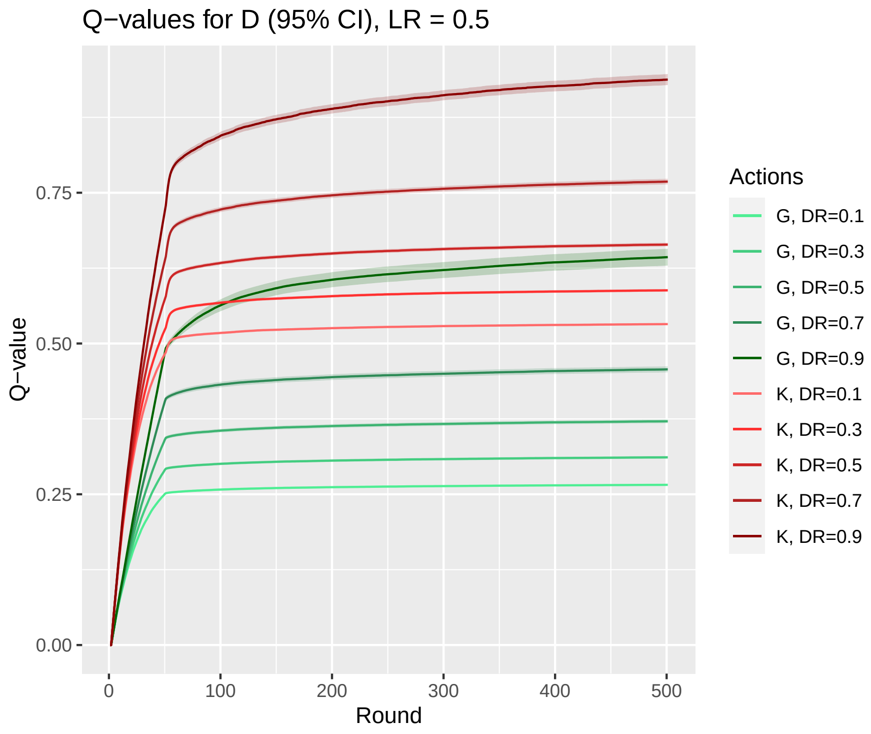

(b) Player D

Figure 3: Case 1 resulting strategies with learning rate of 0.5 and varying discount rates.

4 Learning rate = 0.7

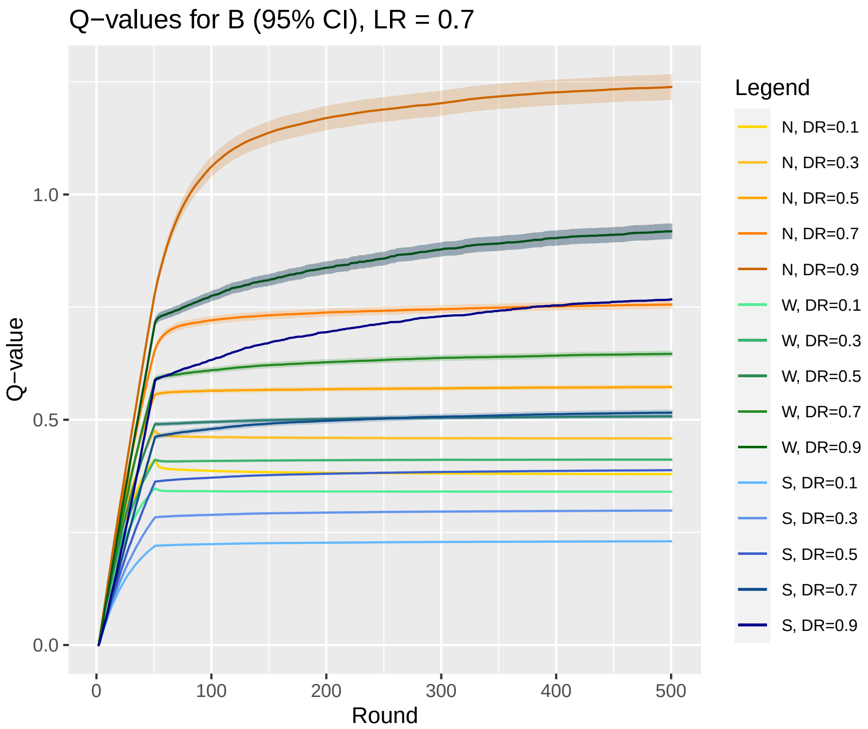

(a) Player B

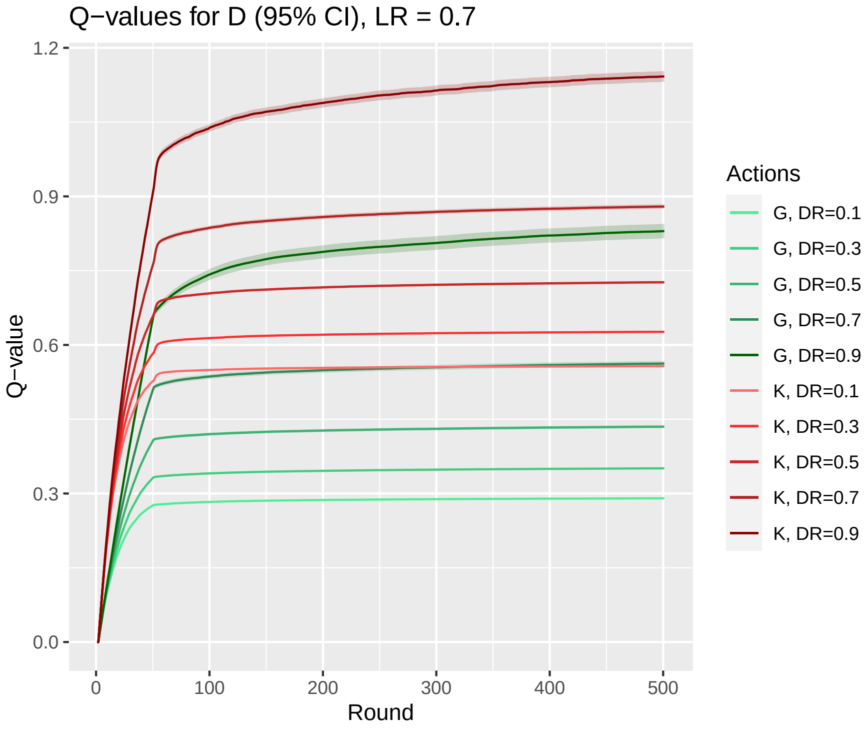

(b) Player D

Figure 4: Case 1 resulting strategies with learning rate of 0.7 and varying discount rates.

5 Learning rate = 0.9

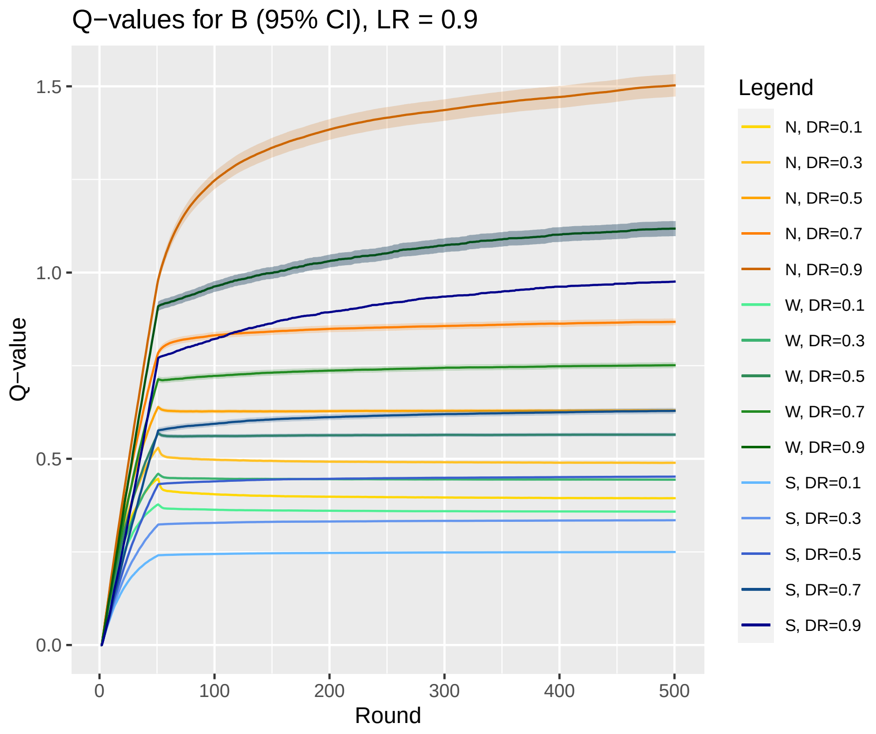

(a) Player B

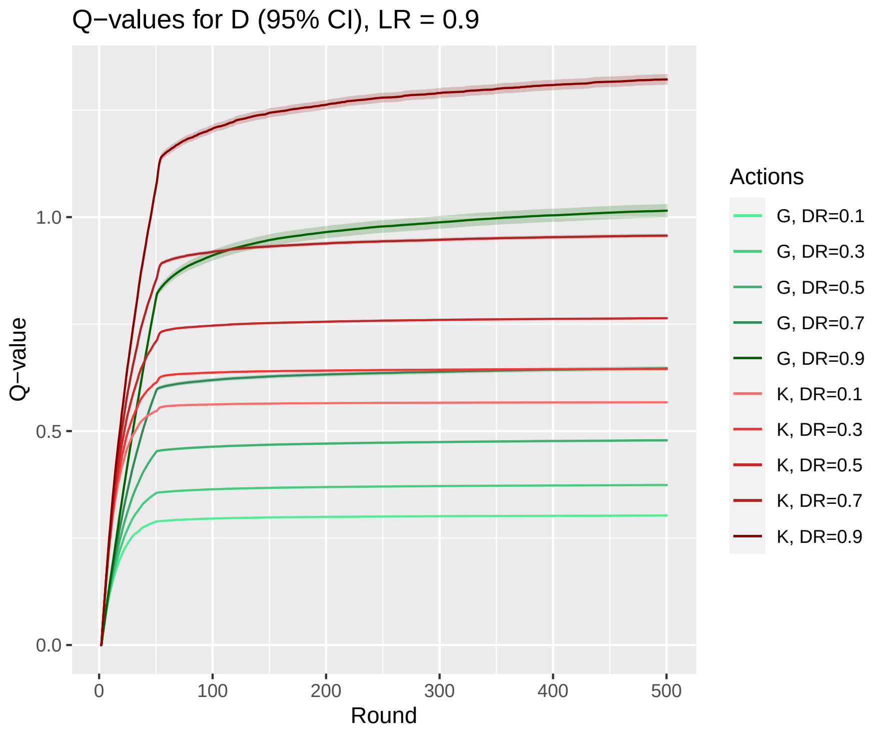

(b) Player D

Figure 5: Case 1 resulting strategies with learning rate of 0.9 and varying discount rates.
