# Supplementary material for: Maynard Smith revisited: A multi-agent reinforcement learning approach to the coevolution of signalling behaviour
Source: PLoS Comput Biol. 2025 Aug 26;21(8):e1013302. doi: 10.1371/journal.pcbi.1013302 (PMC12440204; doi:10.1371/journal.pcbi.1013302)
Supplement: S3 Appendix — (PDF) [file pcbi.1013302.s003.pdf]

S3 Appendix: Results with static agents

1 Case 1: $U = 0.2, V = 0.2, r = 0.5$

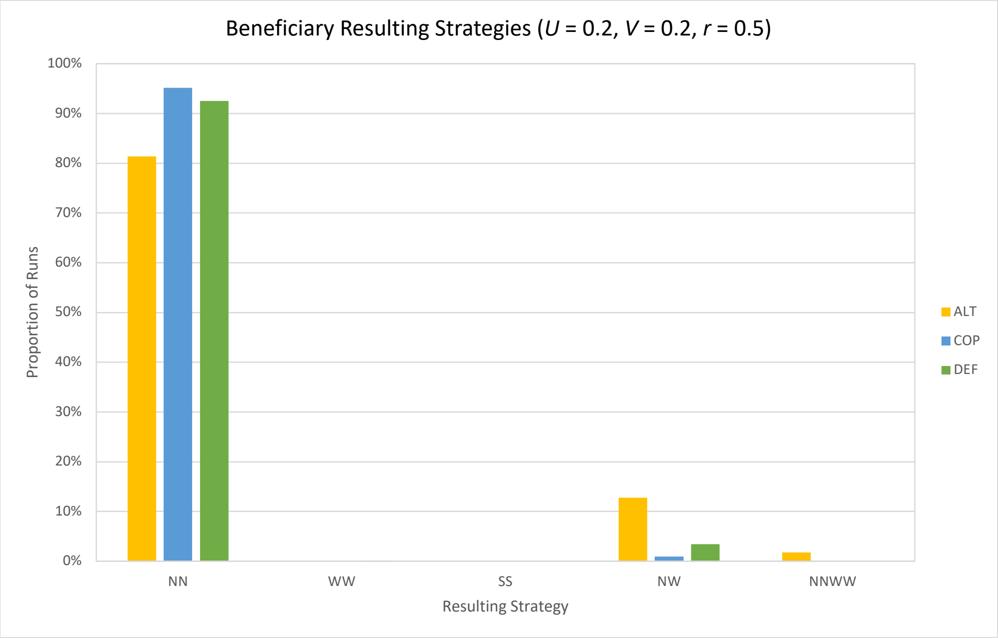

(a) Player B

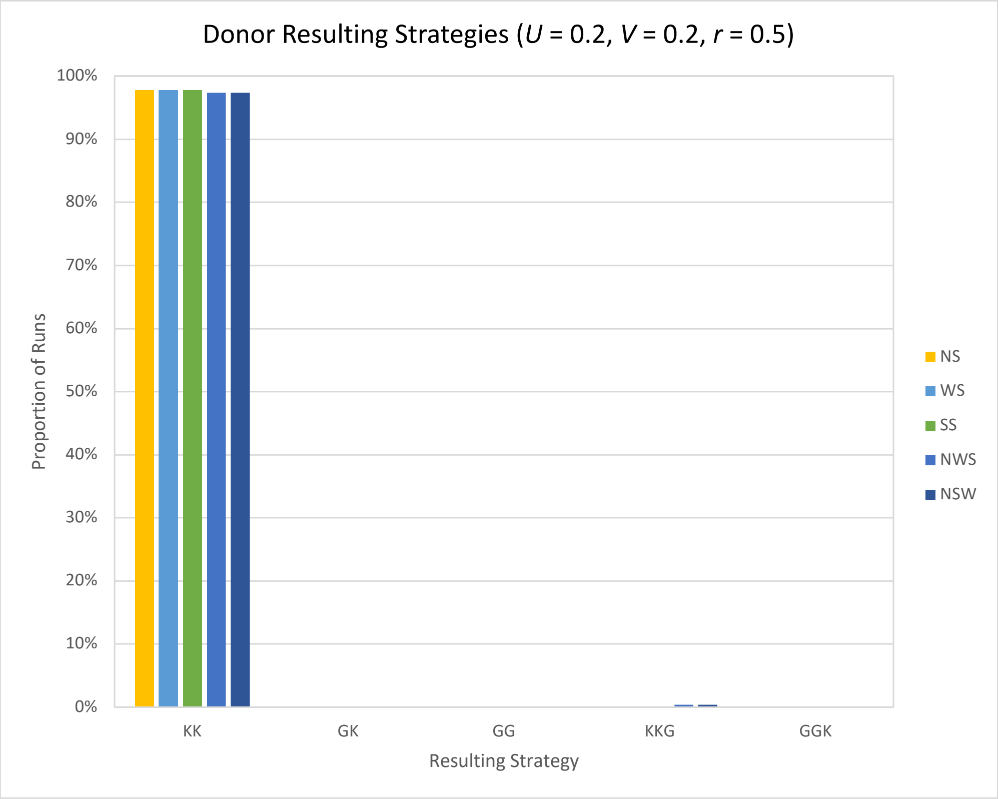

(b) Player D

Figure 1: Case 1 resulting strategies with static opponents.

2 Case 2: $U = 0.9, V = 0.1, r = 0.8$

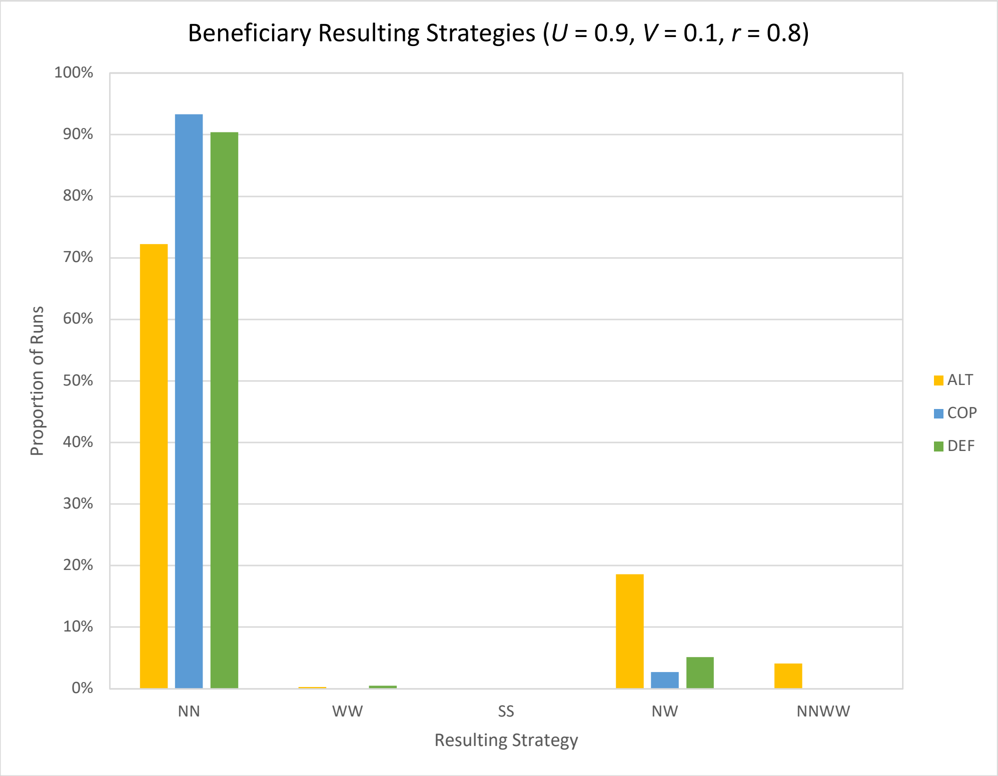

(a) Player B

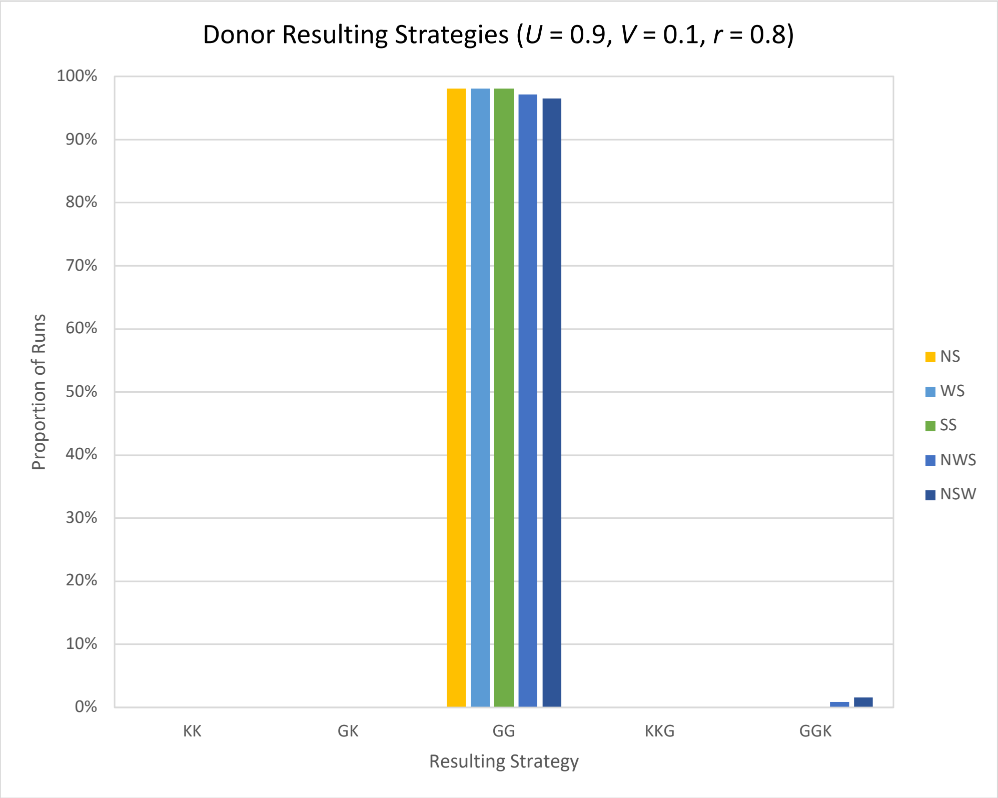

(b) Player D

Figure 2: Case 2 resulting strategies with static opponents.

3 Case 3: $U = 0.95, V = 0.75, r = 0.9$

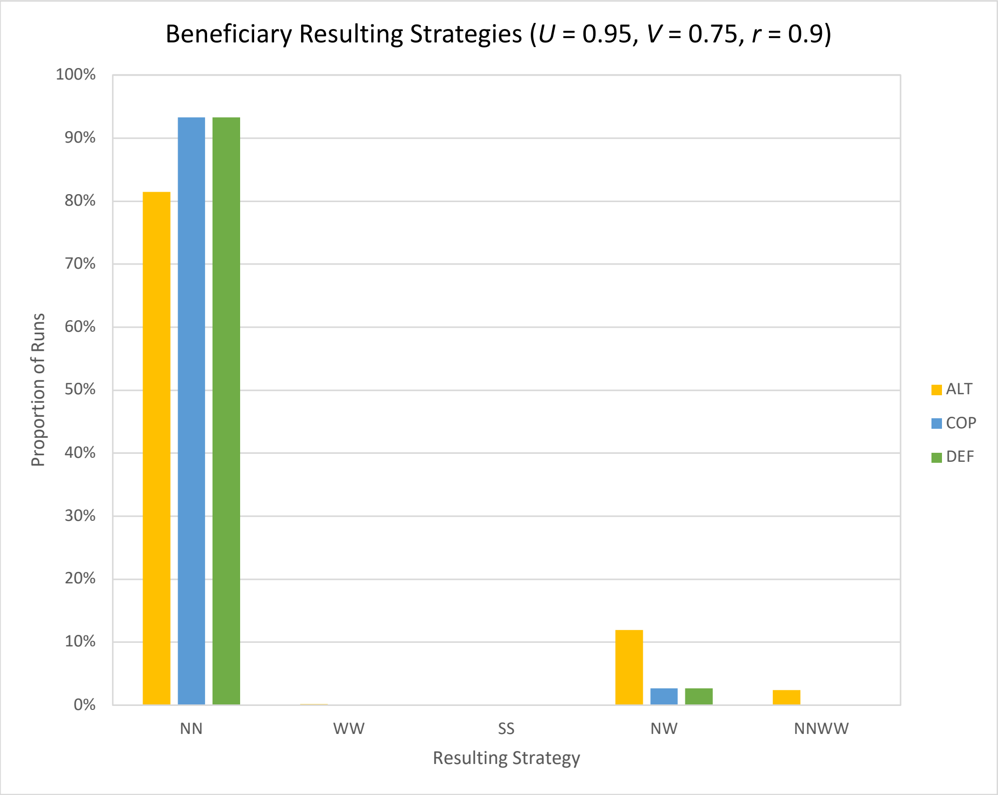

(a) Player B

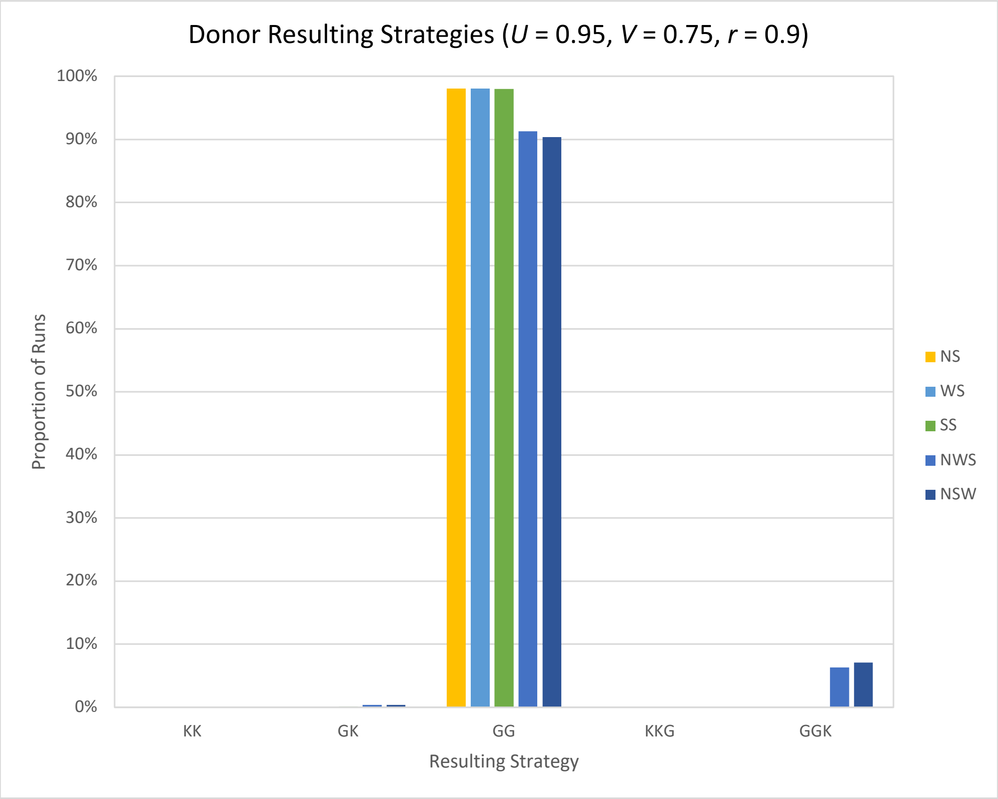

(b) Player D

Figure 3: Case 3 resulting strategies with static opponents.
