# Supplementary material for: Maynard Smith revisited: A multi-agent reinforcement learning approach to the coevolution of signalling behaviour
Source: PLoS Comput Biol. 2025 Aug 26;21(8):e1013302. doi: 10.1371/journal.pcbi.1013302 (PMC12440204; doi:10.1371/journal.pcbi.1013302)
Supplement: S5 Appendix — (PDF) [file pcbi.1013302.s005.pdf]

S5 Appendix: Results with varying exploration

By comparing the effect on the learned Q-values of different exploration lengths and values of ϵ, we can see that the length of initial exploration determines the resulting Q-values to a greater extent than ϵ. We therefore include graphs for three different exploration lengths, comparing values of ϵ; additionally, we include one example graph in each case of ϵ ($\epsilon = 1/N$).

1 Case 1: $U = 0.2, V = 0.2, r = 0.5$

1.1 25 rounds of exploration

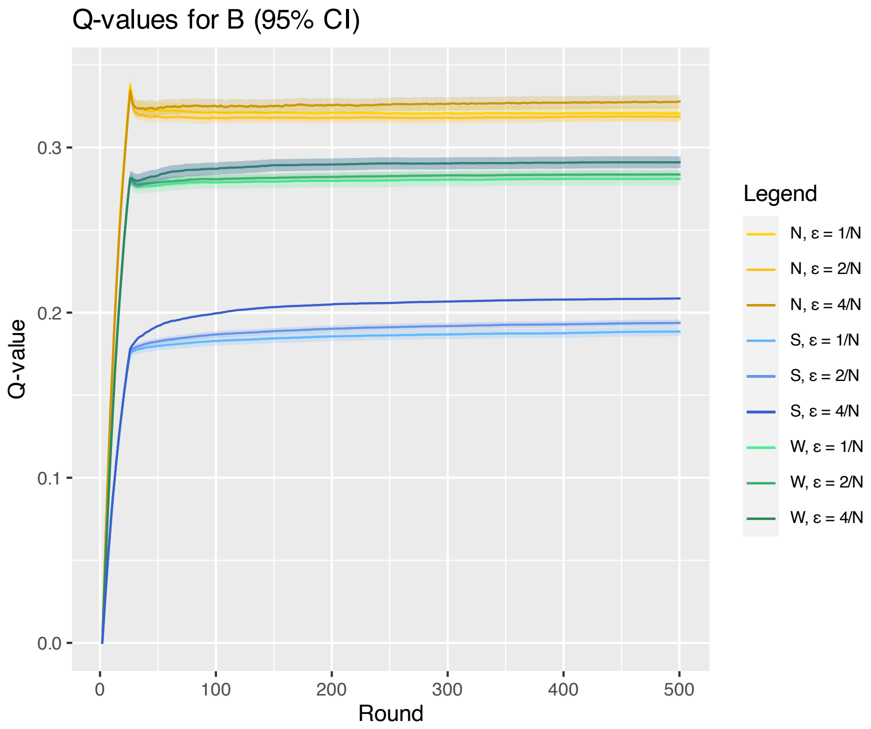

(a) Player B

Figure 1: Q-values Case 1, with an exploration period of 25 rounds.

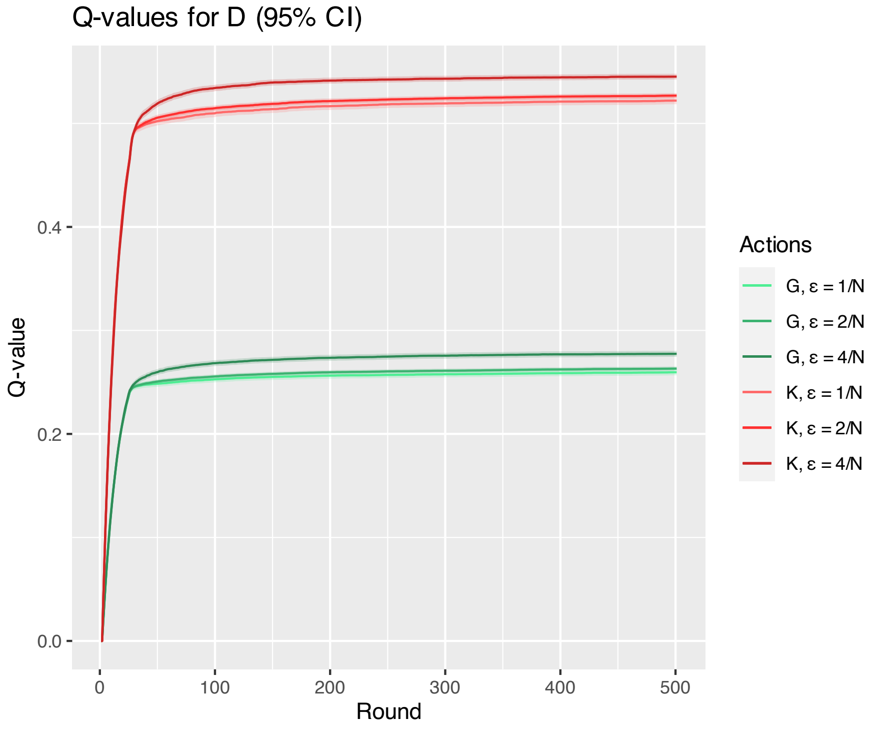

(b) Player D

Figure 1: Q-values Case 1, with an exploration period of 25 rounds (continued).

1.2 50 rounds of exploration

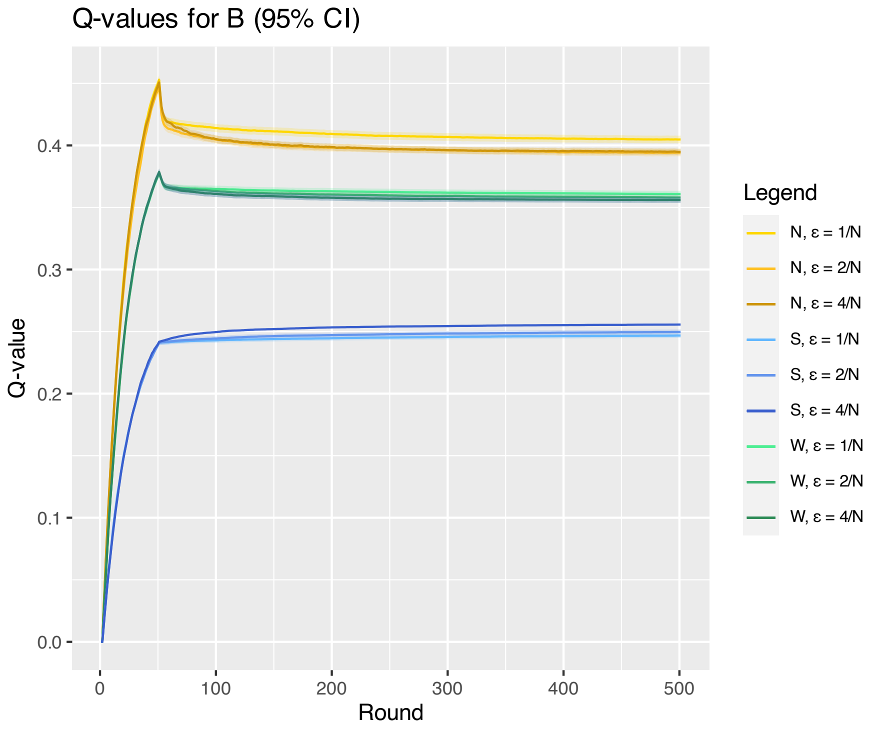

(a) Player B

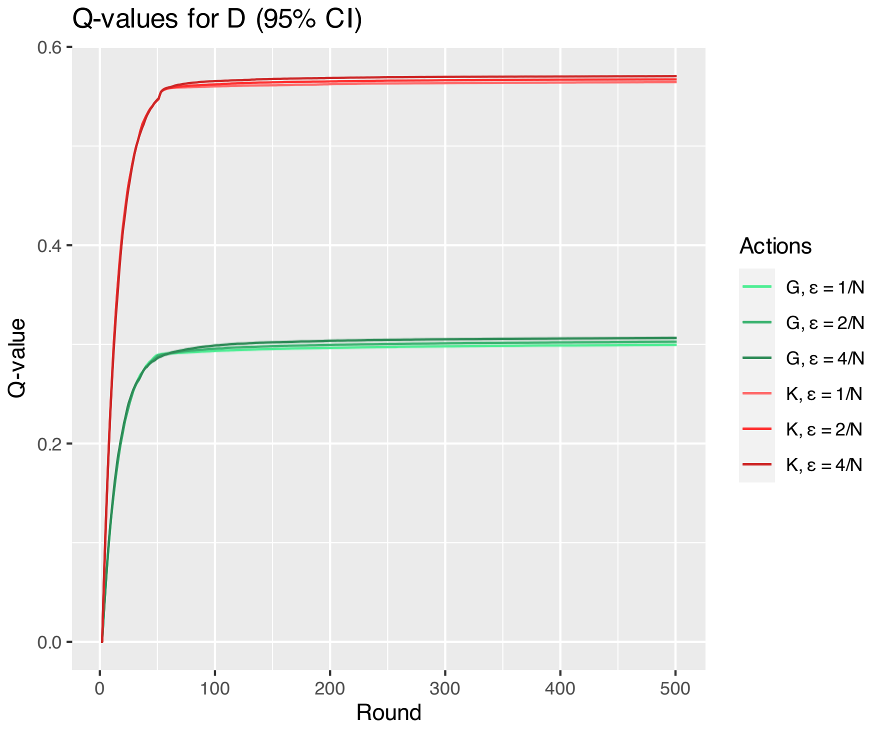

(b) Player D

Figure 2: Q-values Case 1, with an exploration period of 50 rounds.

1.3 100 rounds of exploration

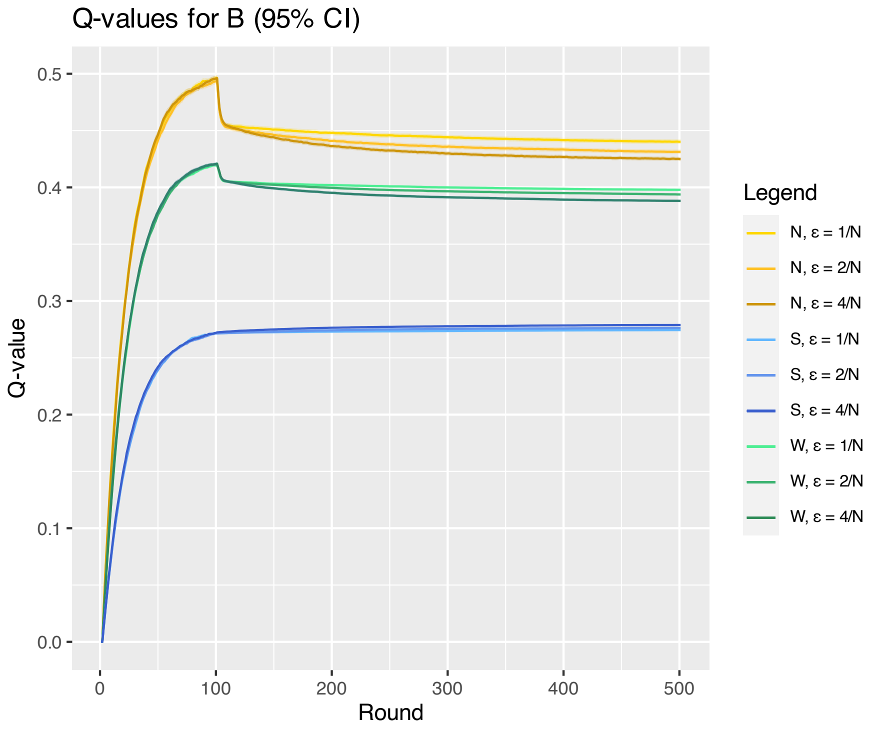

(a) Player B

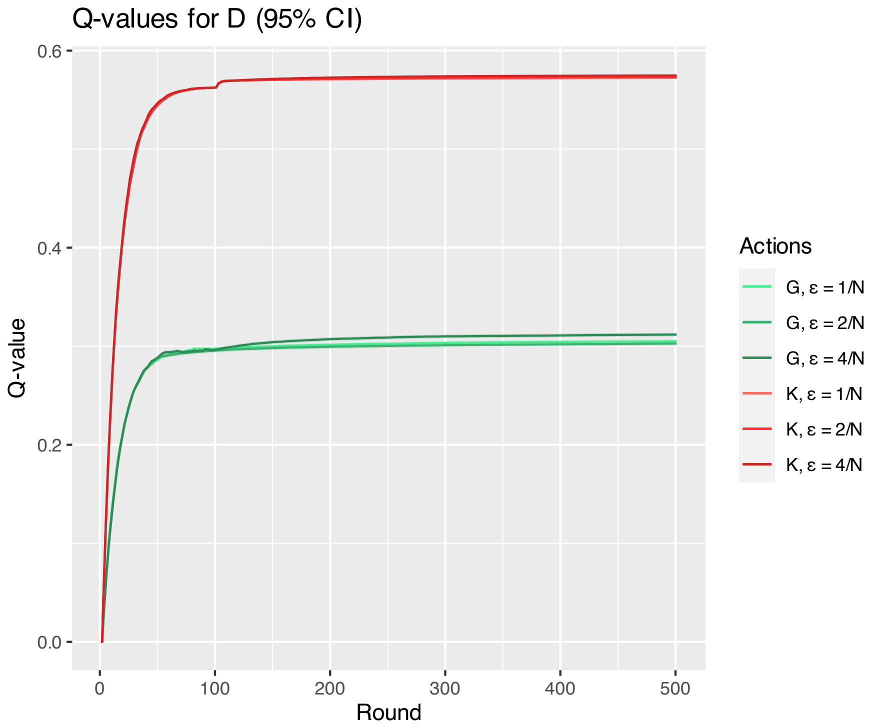

(b) Player D

Figure 3: Q-values Case 1, with an exploration period of 100 rounds.

1.4 $\epsilon = 1/N$

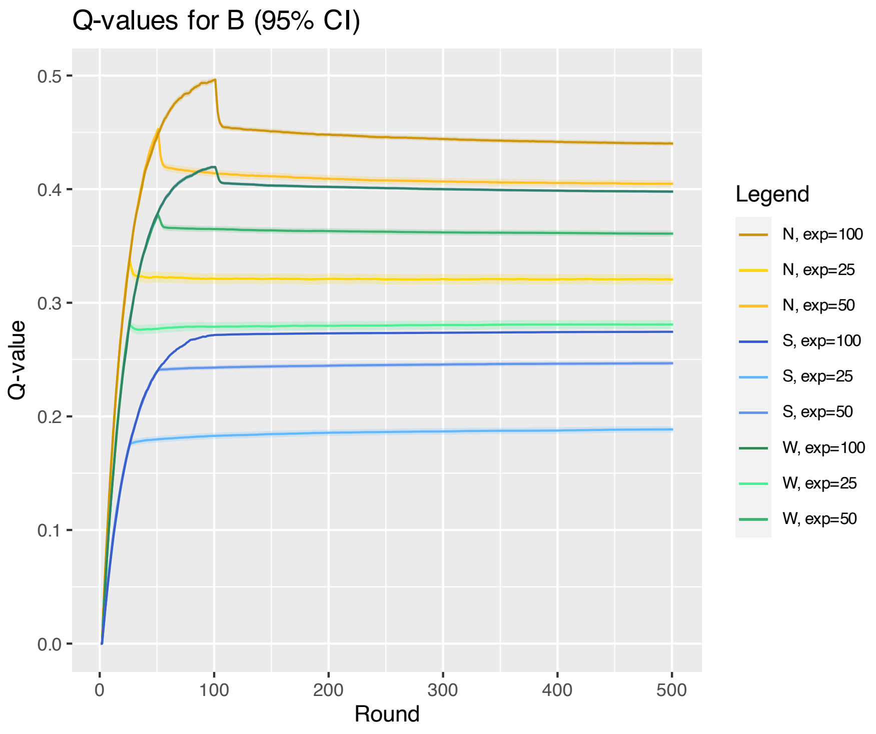

(a) Player B

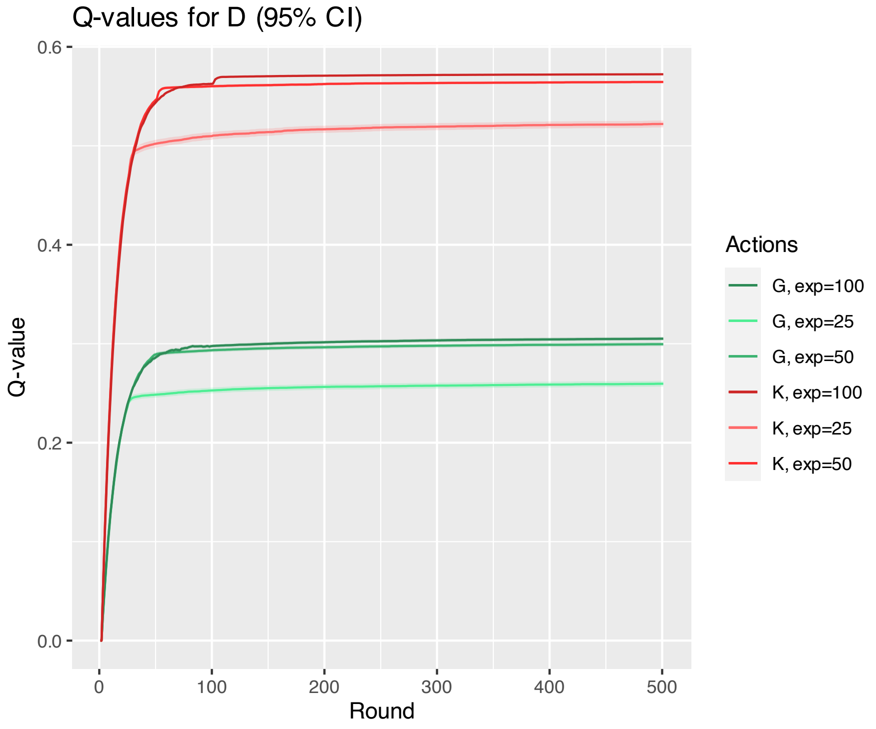

(b) Player D

Figure 4: Q-values Case 1, with $\epsilon = 1/N$.

2 Case 2: $U = 0.9, V = 0.1, r = 0.8$

2.1 25 rounds of exploration

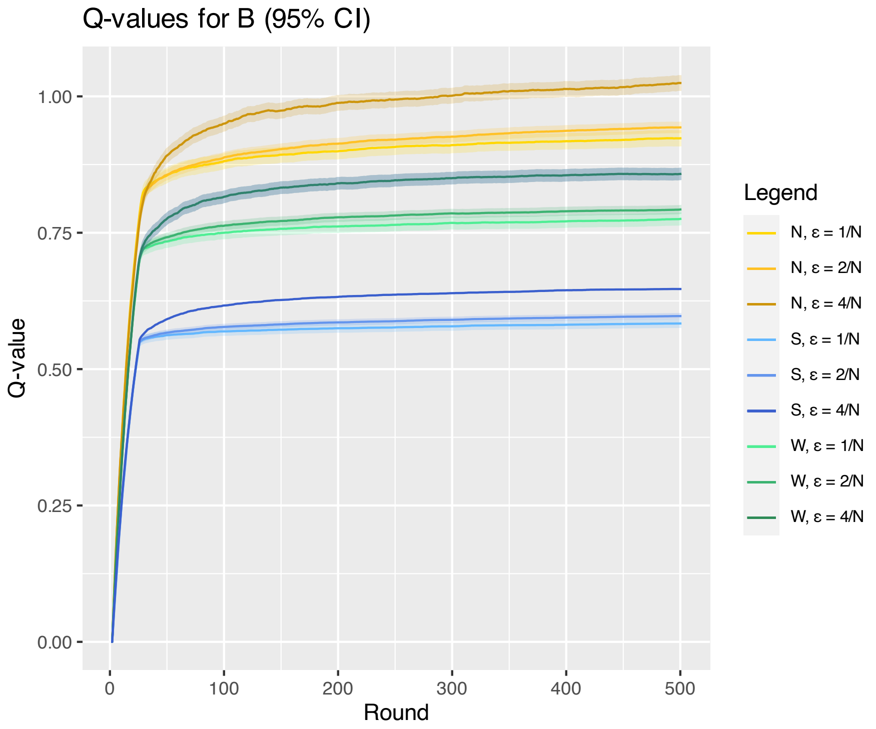

(a) Player B

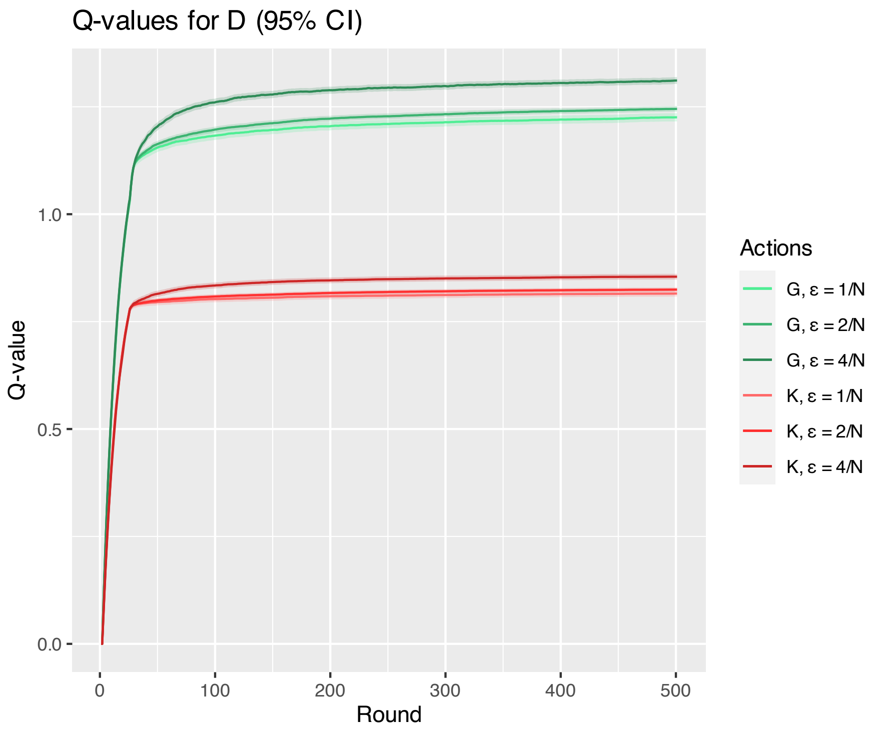

(b) Player D

Figure 5: Q-values Case 2, with an exploration period of 25 rounds.

2.2 50 rounds of exploration

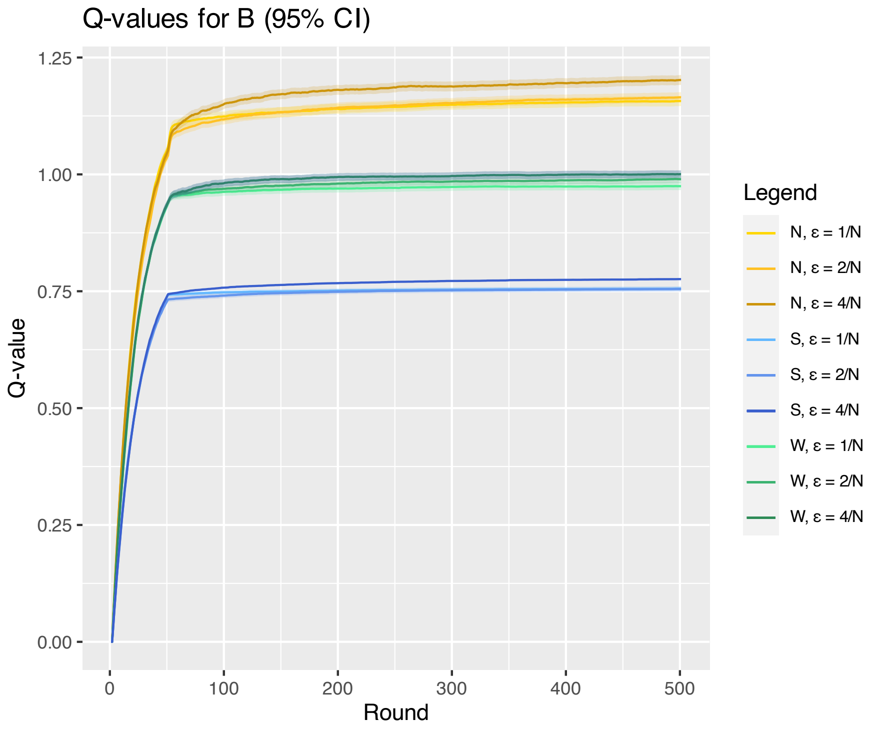

(a) Player B

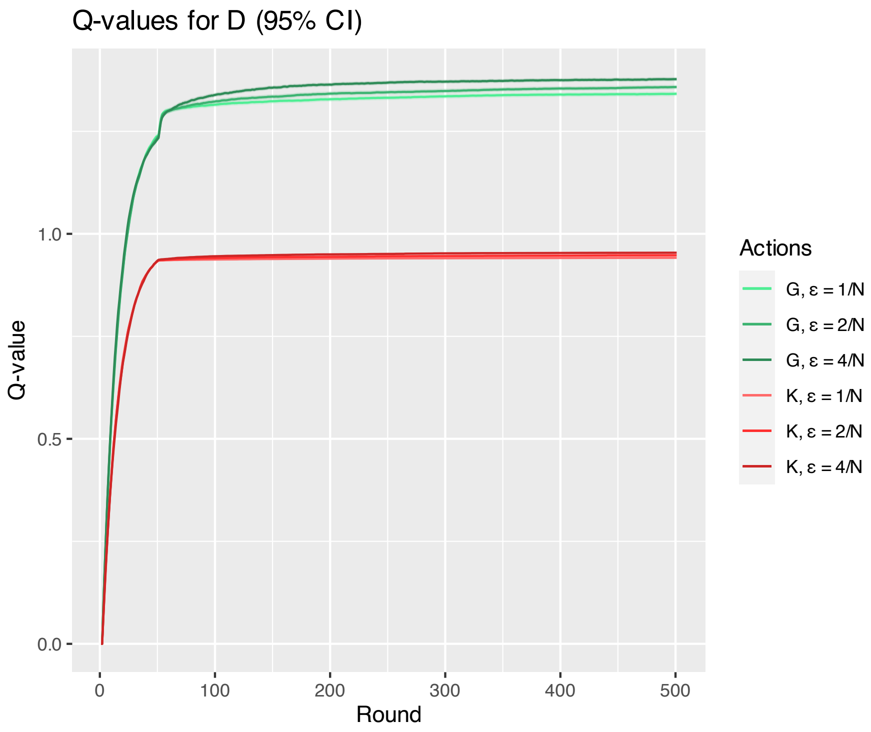

(b) Player D

Figure 6: Q-values Case 2, with an exploration period of 50 rounds.

2.3 100 rounds of exploration

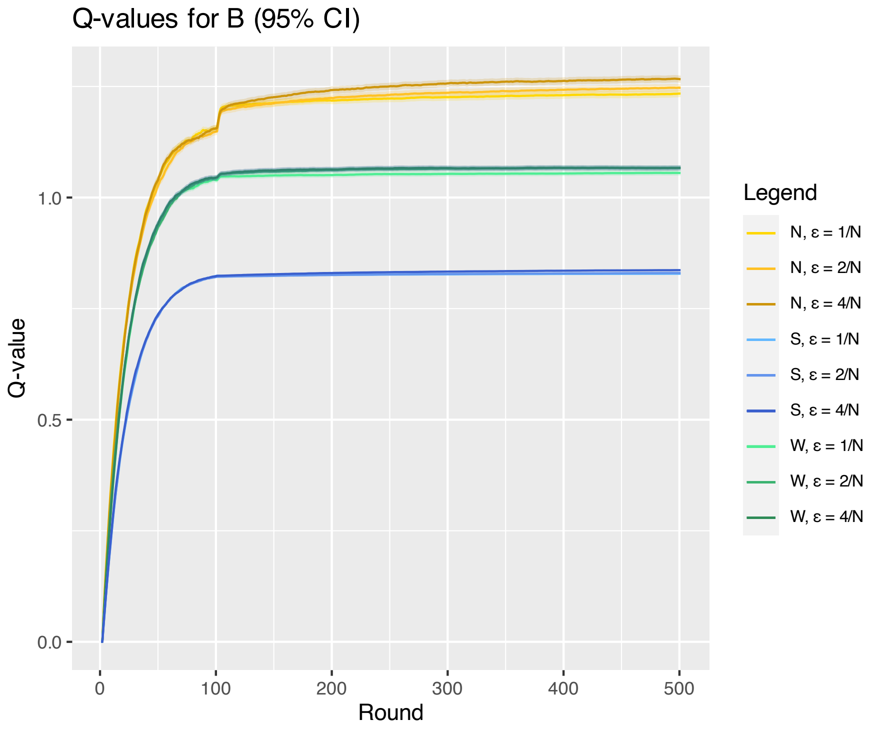

(a) Player B

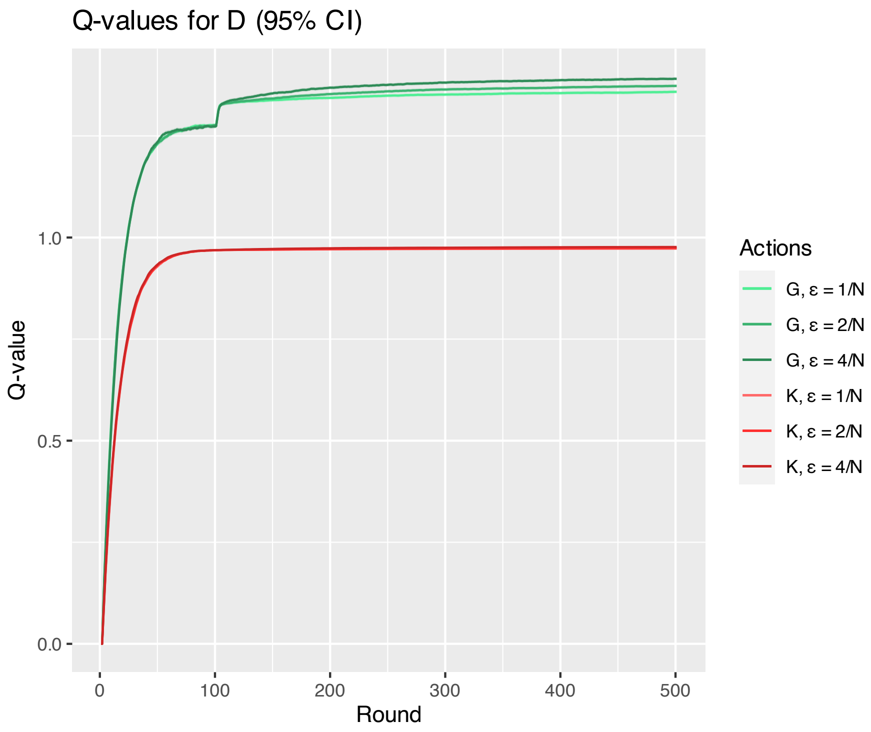

(b) Player D

Figure 7: Q-values Case 2, with an exploration period of 100 rounds.

2.4 $\epsilon = 1/N$

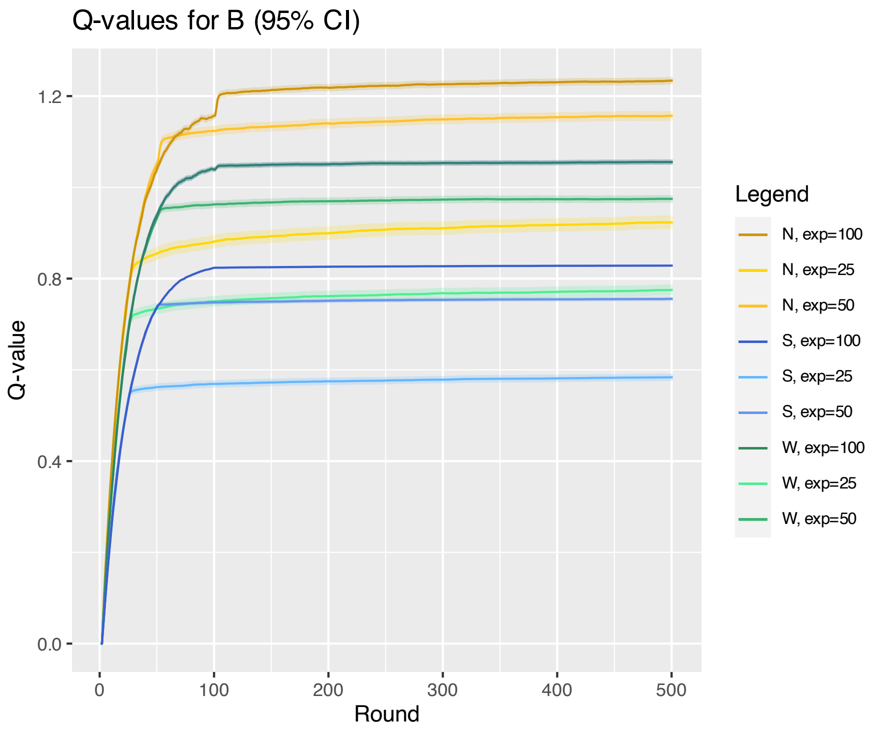

(a) Player B

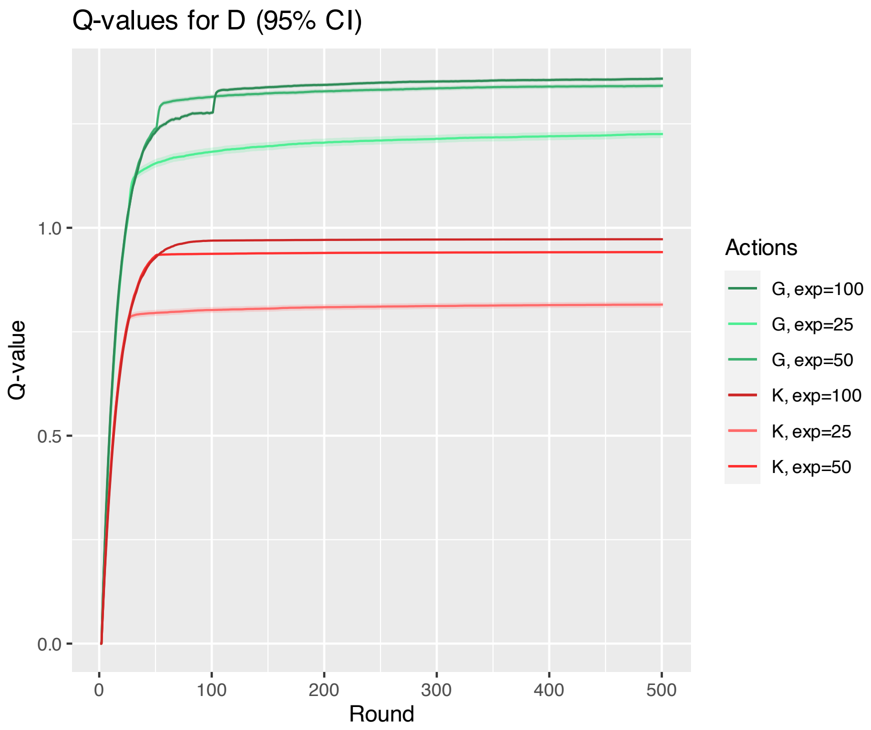

(b) Player D

Figure 8: Q-values Case 2, with $\epsilon = 1/N$.

3 Case 3: $U = 0.95, V = 0.75, r = 0.9$

3.1 25 rounds of exploration

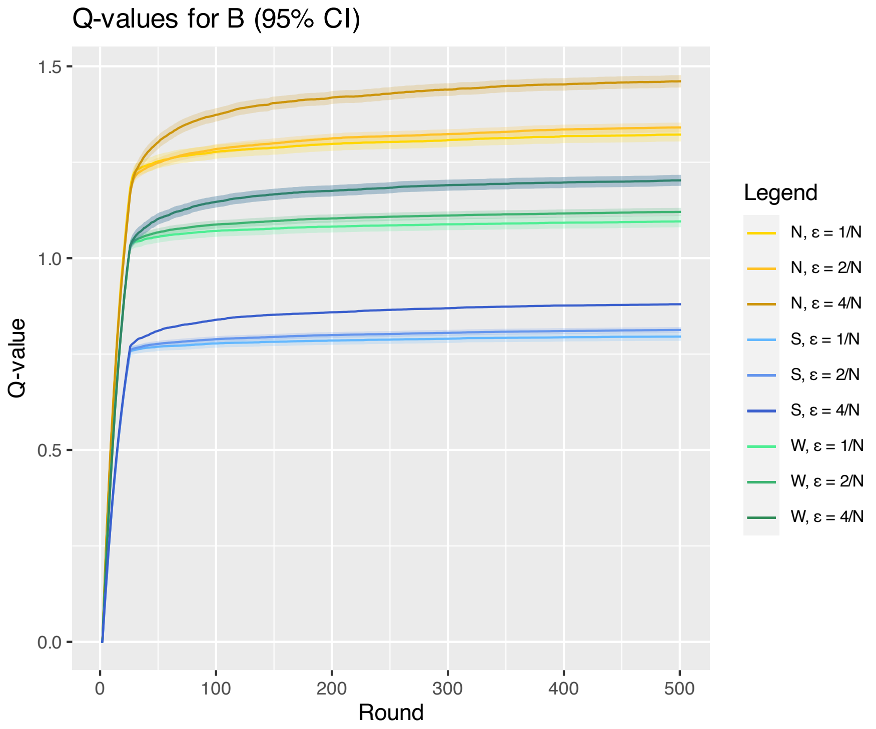

(a) Player B

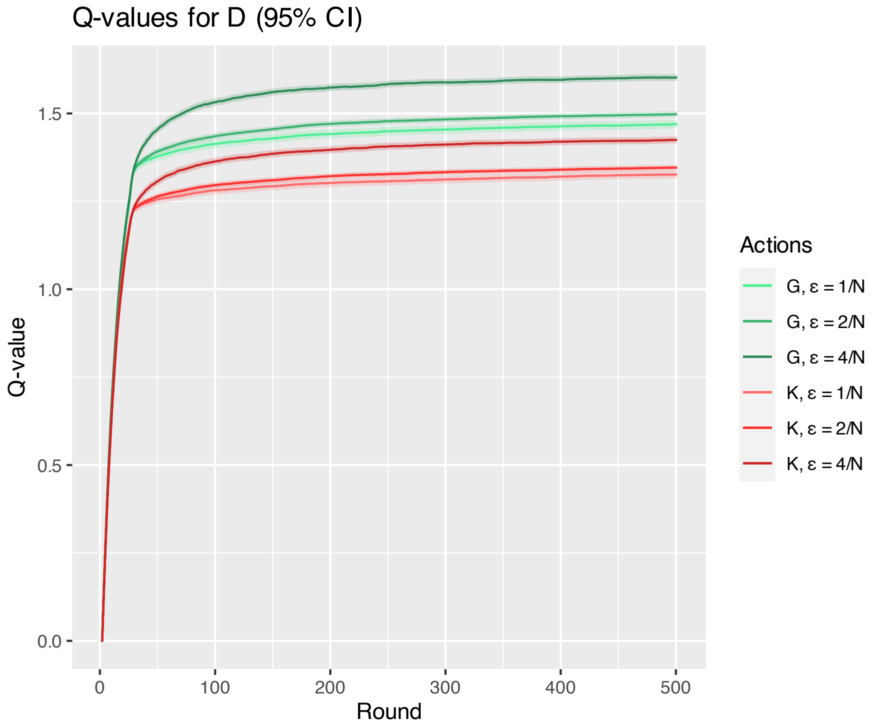

(b) Player D

Figure 9: Q-values Case 3, with an exploration period of 25 rounds.

3.2 50 rounds of exploration

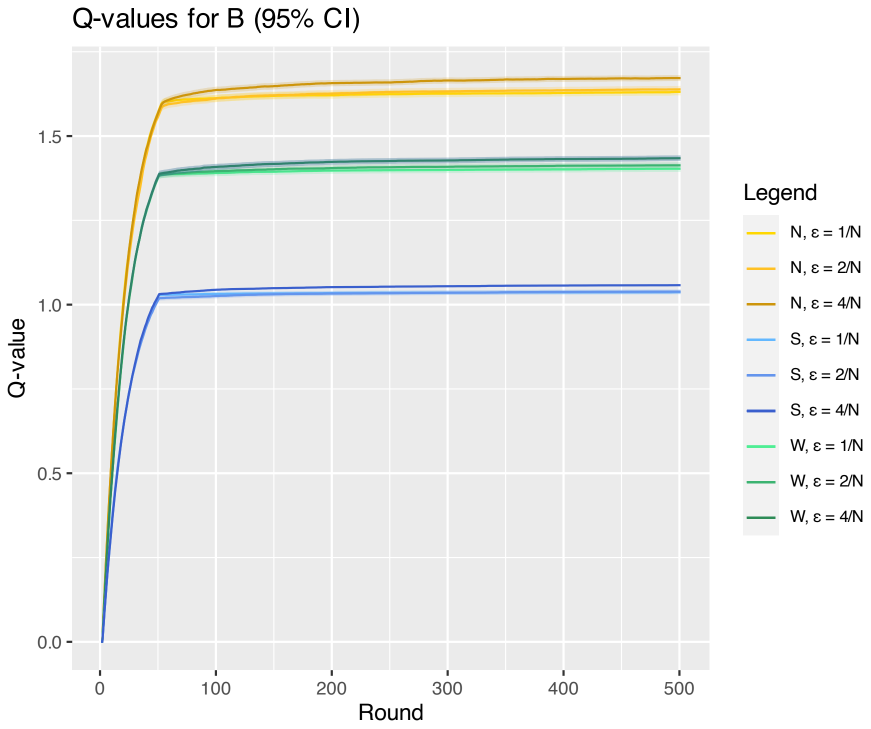

(a) Player B

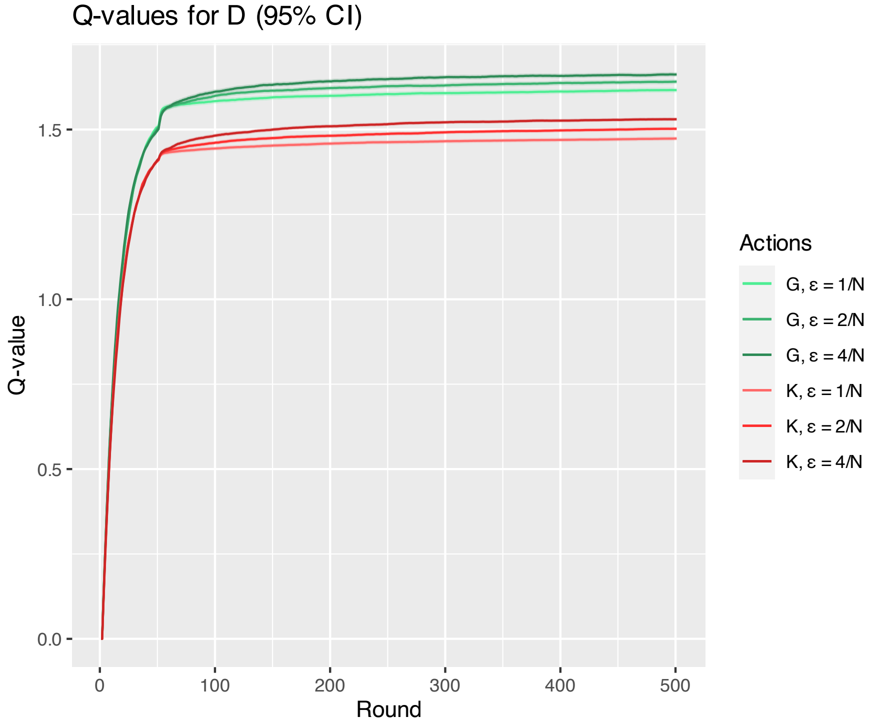

(b) Player D

Figure 10: Q-values Case 3, with an exploration period of 50 rounds.

3.3 100 rounds of exploration

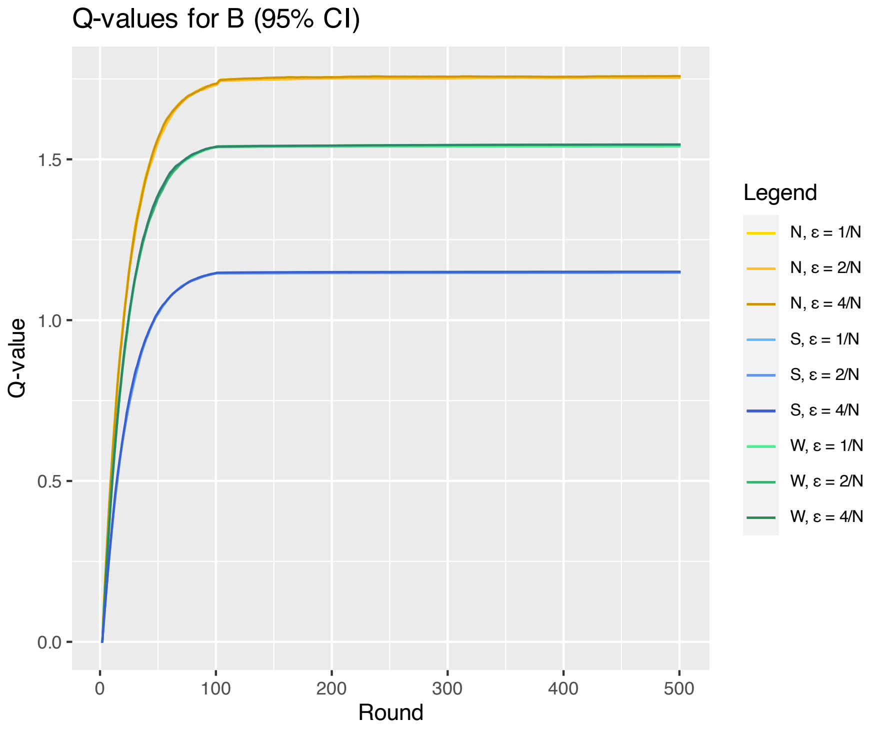

(a) Player B

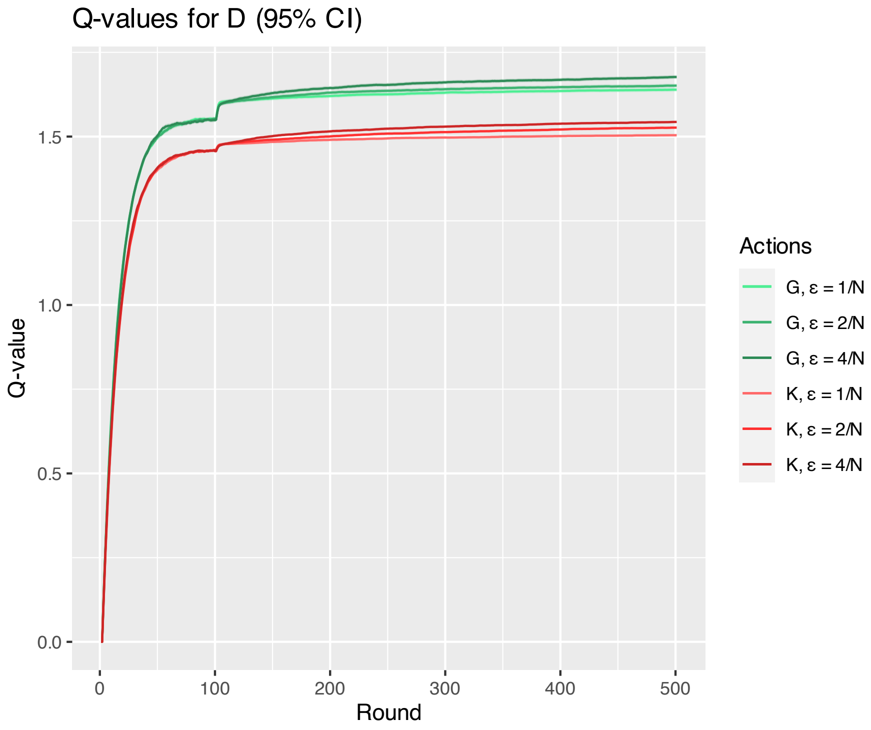

(b) Player D

Figure 11: Q-values Case 3, with an exploration period of 100 rounds.

3.4 $\epsilon = 1/N$

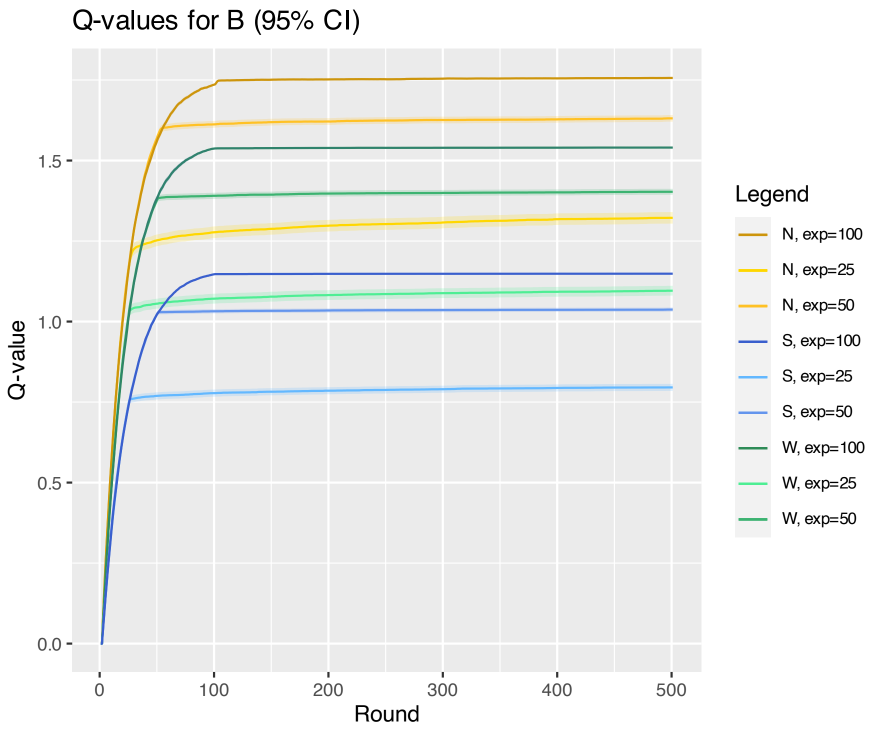

(a) Player B

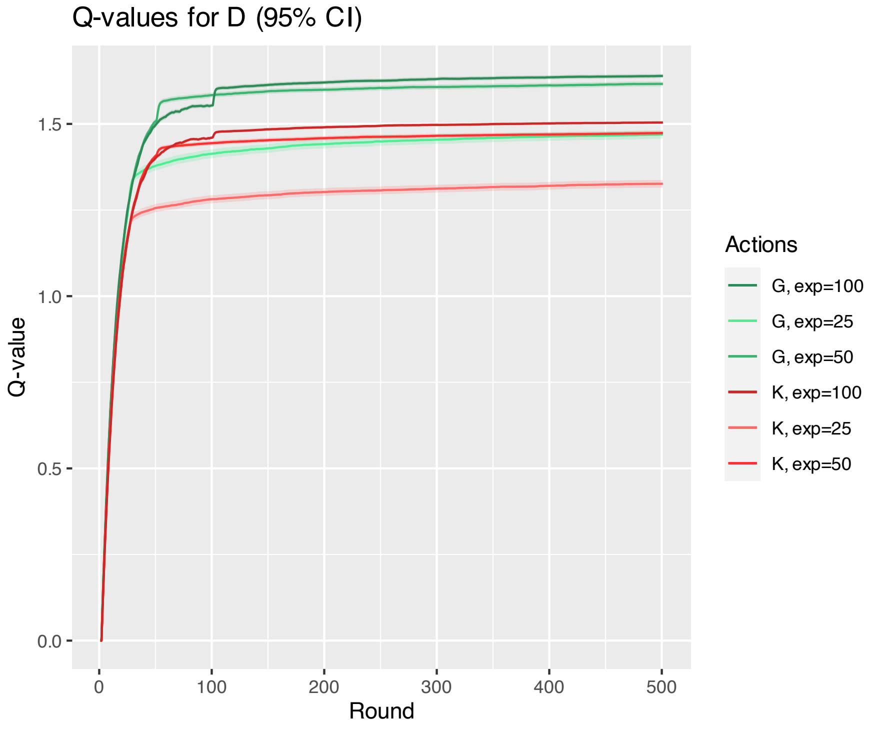

(b) Player D

Figure 12: Q-values Case 3, with $\epsilon = 1/N$.
