# Supplementary material for: Maynard Smith revisited: A multi-agent reinforcement learning approach to the coevolution of signalling behaviour
Source: PLoS Comput Biol. 2025 Aug 26;21(8):e1013302. doi: 10.1371/journal.pcbi.1013302 (PMC12440204; doi:10.1371/journal.pcbi.1013302)
Supplement: Attachment — Submitted filename: Review_of_PLOS_Article.pdf [file pcbi.1013302.s006.pdf]

1 Summary of Article

The paper aims to highlight the strengths offered by reinforcement learning (RL) in modelling adaptive agents through demonstrating deviations from established results in signalling. Additionally, the authors claim that RL offers two key advantages through its interpretability and flexibility which ultimately help in understanding biological evolution, in this case signalling, without pre-specifying the strategies that agents can employ.

The paper examines the learned behaviour of two Q-learning agents repeatedly playing a signalling game against each other called the Philip Sidney game. For the entire duration of play, one agent is assigned the role of the Beneficiary and the other the Donor. The Beneficiary has a p chance of being in a *thirsty* state and a $1-p$ chance of being in a *not thirsty* state and can choose whether to display a costly signal or not after observing their state. The Donor, who observes only the potential signal of the Beneficiary, must decide whether to give water or not.

When agents' payoffs do not consider the payoffs of their interaction partner, it is clear that the Donor would never give water. Maynard Smith showed that when the interaction partners are related (with strength of relation $rin[0,1]$, and therefore each player's utility is their personal utility plus r times their partner's utility, honest signalling and providing water in response to a signal is evolutionarily stable. This paper, however, considers what happens under simultaneous play instead of sequential play, referring to examples in the animal kingdom where benefit is given without prior signalling.

The authors show that, when the Donor's strategy cannot be conditioned on receiving a signal from the Beneficiary and the two agents are related, the dominant strategy of the Beneficiary is not to signal. Then the authors derive a threshold on r as a function of various other parameters beyond which the Donor has a dominant strategy of donating and before which has one of not donating. This threshold is visualised in Figure 2.

The agents in the paper take actions via policies updated through Q-learning, with the action space of the Beneficiary augmented with a more costly and less costly signal option as well as not signalling at all. The state of the agents at each round of play is the action-tuple of both agents from the previous round, and the learning rate is initially fixed at 1 for 50 rounds before decaying in the subsequent 450 rounds.

The paper then runs the model with different parameter and hyper-parameter values as well as replacing one learning agent with a fixed-policy agent, observing in each case how often each action is taken by the agents and noting any cyclical behaviour that occurs. The authors show that when the parameters are close to the previously derived threshold, the optimal strategy is learned less often for both agents.

The authors conclude that multi-agent RL can be used to observe strategies that emerge through co-evolution and suggest areas of further exploration.

2 Strengths

Applying RL to understand biological phenomena and its connection to evolutionary learning is an interesting endeavour. The evolution of signalling is a particularly complex topic and the literature review provided in the introduction of the paper motivates further study in the topic.

Figure 2 showing the threshold of which strategies are evolutionarily stable help in identifying regions of interest for further study. This serves as a good motivation for the subsequent choice of parameter combinations to study. Additionally, the simulations are comprehensive and the supplementary information provides a series of robustness checks by showing results for a wide range of hyperparameters.

3 Major Concerns

While the paper aims to offer insight into the co-evolution of signalling in the Philip Sydney game, the authors make a number of decisions that undermine such goals:

C1: Lack of conditional strategies. First and foremost: the authors explicitly choose "not to assume conditionality" of resource transfers on signals. While I may be mistaken on how to interpret this, my understanding of this decision is that the agents in the paper are no longer playing sequentially, where the Donor's action is taken in response to the Beneficiary's action, but simultaneously. This has a number of implications: as the authors show, it is now dominant for the Beneficiary not to signal and the Donor therefore also has a dominant strategy. Now that both agents have a dominant strategy, the "co-evolution" focus of the paper is, in my opinion, critically undermined. Co-evolution is interesting because it often leads to complex dynamics containing multiple equilibria among which it is not clear which will be arrived at even if dynamics are deterministic. When there is only one equilibrium, and the strategies involved are dominant through iterated elimination as the authors show, it is not surprising that the dominant strategy is learned.

A by-product of the choice not to assume conditionality is that the very meaning of a "signal" is not clear. The aim of the signal in the sequential game is to make the Donor aware of information relevant to their current decision. However, in the simultaneous game, the Donor has no chance to get such information. In the MARL experiments of the paper, the "thirsty" property is determined randomly with probability p and is independent of previous rounds and so, again, any action by the Beneficiary cannot tell the Donor how to act, either now or in the future.

C2: Insufficient clarity in critical points. The second concern I have regarding the paper is related to the first: while the text is generally well written, some key concepts or results in the paper are explained using ambiguous

wording or are otherwise slightly confusing, requiring significant subjective interpretation by the reader. The two most pressing examples are the previously mentioned choice to not "assume conditionality", an explanation of which would be key to understanding the model itself, and the repeated use of the phrase "reinforcement learning does not require a predefined set of strategies" or a similar one. While an informed reader can guess what the authors intend by this, its literal reading, that reinforcement learning (RL) does not require specification of a strategy space, is simply not correct. Given that this phrase is used throughout the paper in order to justify RL as the mechanism of strategic adaption, the correct interpretation must be clear to the reader.

C3: Insufficient justification for use of RL. The paper aims to show that MARL (multi-agent reinforcement learning) can be used to gain insight into "the underlying mechanics of learning over generations", citing two key advantages of MARL as interpretability and the "predefined strategies" point. I believe that the significance of these advantages is overstated, that MARL makes more sense from an engineering perspective, and therefore that other strategy adaption mechanics should be explored.

In individual learning models, as this paper presents, RL allows agents to learn through trial and error and individual rewards. As an engineering tool, RL allows engineers to design agents that maximise long term rewards through exploration of the strategy space. I don't believe this is a particularly realistic way to model strategic adaption in an evolutionary biology sense, where populations of agents *are* present and may be large (effectively infinite as in replicator-mutator dynamics) or small (e.g. agent-based models with weak selection). As is stated in the paper, it is true that in these models agents' strategies are "predetermined" and change from one to another in a discontinuous way, but this argument also holds true for Q-learning: as soon as the Q-value for one action is larger than another then this action is chosen w.p. $1 - \epsilon$. Additionally, while Q-values are directly interpretable as the quality of a certain action in a certain state, the average utility of an individual playing strategy S can also be interpreted as such in a model with "predetermined" strategies. Finally, when using a technique such as adaptive dynamics, the probabilities of playing a certain strategy in a mixed strategy set can be updated in a continuous way such that there is no discontinuous switch between strategies. As such, I believe that the use of MARL is currently not sufficiently justified such that the entire paper centres on its use due to its comparative lack of biological realism, and lack of clear advantages over other methods in interpretability and flexibility.

C4: Modelling decisions are at odds with readers. Many modelling decisions in the paper do not have a strong enough justification that they are biological plausible. A central part of *PLOS Computational Biology* is in providing insight into the mechanics of biological systems so any abstractions or deviations from biological reality should come with a strong justification in order for the results of the study to remain relevant.

For example, while Q-learning at an individual level has been used to model biological phenomena (see Neftci and Averbeck [2019]), the evolutionary perspective on utility taken by inclusive fitness happens at the level of genes and therefore at a generational timescale. Therefore, the biological plausibility of combining the individual learning of RL agents and the generational adaption of inclusive fitness must be justified.

Another example is found in the choice of a learning rate that is fixed for 50 rounds and then decays over time. While this may lead to learning outcomes that maximise long-term utility, there is no evidence given that this way of modelling agents using Q-learning leads to realistic dynamics either in terms of what is learned or the learning trajectories themselves. When one of the key claims of the paper is offering insight into a biological system through understanding its mechanics and the interpretability of the method, the ability of this method to accurately model such a system is important.

4 Conclusion

Despite the valuable goals of the paper in understanding signalling from a mechanistic perspective with reinforcement learning, the modelling decisions made by the authors are often unclear or have poor biological justification. Furthermore, the decision to have agents play simultaneously leads to results that are difficult to interpret in the context of signalling, where actions are typically taken in response to signals or the lack thereof. Finally, Q-learning does not clearly provide benefits over other methods that allow for the adoption of mixed-strategies, and its combination with inclusive fitness does not serve the biological plausibility of the model presented.

References

Emre O. Neftci and Bruno B. Averbeck. Reinforcement learning in artificial and biological systems. *Nature Machine Intelligence*, 1(3):133–143, March 2019. ISSN 2522-5839. doi: 10.1038/s42256-019-0025-4. URL `https://www.nature.com/articles/s42256-019-0025-4`.
