# Supplementary material for: Maynard Smith revisited: A multi-agent reinforcement learning approach to the coevolution of signalling behaviour
Source: PLoS Comput Biol. 2025 Aug 26;21(8):e1013302. doi: 10.1371/journal.pcbi.1013302 (PMC12440204; doi:10.1371/journal.pcbi.1013302)
Supplement: Attachment — Submitted filename: Response to Reviewers.docx [file pcbi.1013302.s007.docx]

**Rebuttal Letter**

The authors would like to thank the reviewers for their fair reviews and detailed list of constructive suggestions. We greatly appreciate the opportunity to resubmit our revised paper for your renewed consideration. Below we respond to the reviewers’ comments and point to the corresponding changes we made in the paper. We have inserted in this letter some of the text that has added been to the manuscript, along with line numbers, and have also submitted the new version of the manuscript with all changes highlighted.

## **Reviewer 1**

1. *While the paper argues that MARL can be used to study emergent behaviour without pre-defined strategies, it seems the strategy space is still pre-defined (e.g. in Tables 5 and 6). It seems the difference between MARL and evolutionary game theory methods is more about that in the latter, all the strategies are included in model and are always analysed, while in the former, it might the case that not all strategies might be learned via MARL. Please clarify this.*

We thank the reviewer for this comment, as indeed it was not clear enough what the strategies presented in Tables 5 and 6 represent. In response to the reviewer’s comment, we would like to clarify that through the use of multi-agent reinforcement learning (MARL), specifically models consisting of agents based on Q-learning, the set of strategies is not pre-defined. The strategy space is made up of all possible strategies, and any of these can be learned by the agent in question. We understand how our use of wording could be unclear, so have changed this to instead note that MARL does not require us to constrain the strategy set from the outset. The difference between MARL and evolutionary game theory methods is that the former involves learning: the agents learn through repeated interaction what action and strategy leads to the highest reward. We believe that the role of evolutionary computation models and MARL are complementary. Through evolutionary computation models, it is possible to study the dynamics of a given set of strategies in a population through generations and observe which emerge as stable. Through MARL, any strategy can be learned, and we are instead interested in what strategies appear to be optimal through learning. The two methods have differing goals: in comparison to evolutionary game theory methods like replicator dynamics, where the set of strategies is indeed predefined and constrained from the outset and we want to see which are selected, using MARL we can study the possible emergence and establishment of any potential strategy for the given scenario. We hope that this clarification addresses the concerns raised by the reviewer.

Tables 5 and 6 illustrate the strategies most frequently learned through MARL by the *Beneficiary* and *Donor* agents respectively. The strategies are shown in Figures 3 and 4; these accompanying tables provide a brief explanation of what each of the strategies is. This has been clarified both in the body of the text and in the table captions. We have added the text below to clearly convey what is presented in the tables:

Lines 351-354:

“The strategies presented in these tables are those most often learned by the reinforcement learning agents that appear in the corresponding graphs. This is not the full set of possible strategies, as through MARL, any action combination can be learned”

The new captions for Tables 5 and 6 read:

Table 5: “Strategies most often learned by the Beneficiary with parameters S=0.2, V=0.2, r=0.5 (Case 1), see Fig 3.”

Table 6: “Strategies most often learned by the Donor with parameters S=0.2, V=0.2, r=0.5 (Case 1), see Fig 4.”

2. *The paper identifies proactive pro-sociality as the key emergent behaviour. While this is a reasonable conclusion given the simulations, it would be important to consider alternative explanations. For example, what is the role of kin selection in here?*

We agree with the reviewer that we should clearly distinguish between what we identify as emergent behaviour and the underlying mechanisms. Regarding kin selection, this may either be prompted or unprompted. If it is prompted by a signal, this would not fall under the {*Not signal, Give}* equilibrium. If it is unprompted, so does not require a signal, kin selection may indeed sometimes be characterised by this equilibrium. In this case, we would argue that kin selection aligns with proactive prosociality as an observed behaviour – kin selection may be the underlying mechanism, where proactive prosociality is what we observe. It is worth noting that we do not identify proactive prosociality as a causal explanation of the observed behaviour. Instead, we discuss proactive prosociality as what the {*Not signal, Give*} equilibrium may look like in animal behaviour. As we mention in the text, it is a behaviour that has been recorded and studied in multiple animal species.

The role of kin selection is captured by the relatedness coefficient$r$. Kinship does indeed affect the likelihood of cooperation: for the same set of parameters, the *Donor* is more likely to learn to share the resource the higher $r$ is (so the more closely related to the *Beneficiary*). This is illustrated in Fig 2, where the higher the value of $r$, the higher the threshold of the signal cost $c$ above which the equilibrium {*Not signal, Give*} no longer holds. Having said that, the equilibrium is still observed at low values of $r$, meaning that kin selection cannot be the only mechanism. Other potential mechanisms may include social learning or reciprocal altruism, where there is no expectation of immediate reciprocity.

We have added a clarification in the manuscript accordingly to distinguish between the observed behaviour that can be assimilated to the equilibrium we find, in contrast to potential underlying mechanisms.

*Also related to this, there are previous stochastic analyses of the Philip Sidney signalling game which show that a diverse equilibrium points are possible, including those involve providing without signaling, see e.g. "Evolutionary stability of honest signaling in finite populations." 2013 IEEE Congress on Evolutionary Computation. IEEE, 2013. and "Evolution of honest signaling by social punishment." Proceedings of the 2014 annual conference on genetic and evolutionary computation. 2014. This stochastic analysis usually leads to a richer set of possible outcomes (see e.g. "Emergence of cooperation and evolutionary stability in finite populations." Nature 428.6983 (2004): 646-650.) It would be useful to compare MARL with this approach.*

The first paper mentioned by the reviewer was already discussed in the manuscript, and we have now added a more explicit discussion of these stochastic analyses and how they compare to other approaches (lines 72-87). While we already referred to the first paper (*Evolutionary stability of honest signaling in finite populations*) as an example of an analysis of the Sir Philip Sidney game, we did not discuss these approaches in depth as the paper focuses on comparing the findings in finite populations to previous analyses in infinite populations, and looks at the effect of varying population size. Similar to replicator dynamics, these stochastic analyses using finite populations also rely on a predefined set of strategies. In this sense, the lack of predefined strategies through the use of MARL presents a similar advantage over the stochastic analyses mentioned as it does over replicator dynamics.

3. *While it’s good to extend the strategy sets for both donor and beneficial strategies in the paper analysis, it raises the questions if it’s useful to study MARL analysis for the original sets of strategies (for example in the two references above) as the baseline? Otherwise, it would be important to justify why this original setup was not adopted for a MARL analysis (which might allow a more direct comparison?).*

This comment appears to assume that using MARL involves analysing a predefined set of strategies – if we have misunderstood this comment, we would welcome a clarification and would be happy to respond. We have clarified that this is not the case in the response to the first comment above, as well as in the manuscript, as we understand that this was not stated clearly enough; it is not that we have chosen to extend the strategy set beyond those in the papers mentioned, rather that the strategies that we present in the paper are those learned by the reinforcement learning agents.

## **Reviewer 2**

1. ***Lack of conditional strategies****. First and foremost: the authors explicitly choose “not to assume conditionality” of resource transfers on signals. While I may be mistaken on how to interpret this, my understanding of this decision is that the agents in the paper are no longer playing sequentially, where the Donor’s action is taken in response to the Beneficiary’s action, but simultaneously. This has a number of implications: as the authors show, it is now dominant for the Beneficiary not to signal and the Donor therefore also has a dominant strategy. Now that both agents have a dominant strategy, the “co-evolution” focus of the paper is, in my opinion, critically undermined. Coevolution is interesting because it often leads to complex dynamics containing multiple equilibria among which it is not clear which will be arrived at even if dynamics are deterministic. When there is only one equilibrium, and the strategies involved are dominant through iterated elimination as the authors show, it is not surprising that the dominant strategy is learned.*

*A by-product of the choice not to assume conditionality is that the very meaning of a “signal” is not clear. The aim of the signal in the sequential game is to make the Donor aware of information relevant to their current decision. However, in the simultaneous game, the Donor has no chance to get such information. In the MARL experiments of the paper, the “thirsty” property is determined randomly with probability p and is independent of previous rounds and so, again, any action by the Beneficiary cannot tell the Donor how to act, either now or in the future.*

We thank the reviewer for this comment, as we agree that further clarification was needed on this point. What is meant by the statement that we chose “not to assume conditionality” is in fact not that the game is no longer sequential. We do *not* mean that the Donor’s action is not conditional on the Beneficiary’s. In our model, the game remains sequential, and the Donor observes the Beneficiary’s action before taking their own. In previous literature, the potential of an equilibrium where the Beneficiary does not signal and the Donor still gives the resource has been discarded (Huttegger and Zollman, 2010). In this literature, the Donor can only give the resource in the event of a signal by the Beneficiary – the resource cannot be given in the absence of a signal. Therefore, this approach to the Sir Philip Sidney game in previous literature imposes a conditionality on the resource being given: the Beneficiary must have signalled first. As mentioned, this eliminates the possibility of an equilibrium where the Beneficiary does not signal and the Donor gives the resource regardless. What we mean in our paper by choosing “not to assume conditionality” is that we are not discarding the possibility of the {*Not Signal, Give*} equilibrium. The Donor’s *Give* action is not conditional on a Beneficiary’s *Signal*, i.e., the Donor can still choose to *Give* even if the Beneficiary chose to *Not Signal*. That is not to say that the Donor does not choose their action after the Beneficiary. The game remains sequential, and by removing the conditionality assumption we do not discard a possible equilibrium. As mentioned in the text, we chose to remove the conditionality assumption as by removing it we consider the possibility of the {*Not Signal, Give*} equilibrium, which can be assimilated to interactions observed in animal behaviour.

To ensure that it is clear what we mean by removing the assumption of conditionality, the following text has been added to the manuscript:

Lines 109-197:

“By not assuming conditionality on the beneficiary's signal, we are also including the possible action combination of {*Not Signal, Give*} that has been excluded in previous literature. This is not to say that the donor's action is no longer conditional on the beneficiary's. The game remains sequential, and the donor still observes the beneficiary's action before taking their own. The only difference is that whereas following previous literature the donor would not have been able to give the resource in the absence of the signal, we do not impose this condition.”

Lines 434-436:

“The equilibrium shown in this paper is reached when we remove the condition that the resource cannot be given in the absence of a signal. The game remains sequential, with *D* only acting after observing *B*'s action”

2. ***Insufficient clarity in critical points****. The second concern I have regarding the paper is related to the first: while the text is generally well written, some key concepts or results in the paper are explained using ambiguous wording or are otherwise slightly confusing, requiring significant subjective interpretation by the reader. The two most pressing examples are the previously mentioned choice to not “assume conditionality”, an explanation of which would be key to understanding the model itself, and the repeated use of the phrase “reinforcement learning does not require a predefined set of strategies” or a similar one. While an informed reader can guess what the authors intend by this, its literal reading, that reinforcement learning (RL) does not require specification of a strategy space, is simply not correct. Given that this phrase is used throughout the paper in order to justify RL as the mechanism of strategic adaption, the correct interpretation must be clear to the reader.*

A clarification for the conditionality assumption is provided in the previous point, so we will focus on the second phrase mentioned: that reinforcement learning does not require a predefined set of strategies*.* If there are any additional points where the wording remains ambiguous or unclear, we would be grateful if these could be pointed out so that we can provide further clarification in the paper.

Regarding the reviewer’s comment that the notion that “reinforcement learning (RL) does not require specification of a strategy space, is simply not correct,” we would like to point at that we do not claim there is no defined strategy space in reinforcement learning (RL), or more specifically Q-learning. The strategy space is made up of all the possible strategies that could be learned by the players given the set of states and actions and the environment the agent acts in; the strategies that can be learned are only constrained by the action space, which is defined as part of the game. In the paper, we mention that through using RL there is no requirement to use a predefined set of strategies in the analysis; the RL agents are provided a state and a set of possible actions at each turn, and we observe the strategies that emerge when these choices are made over many rounds. The resulting strategies will naturally belong to the strategy space, as this includes all possible strategies that an agent could learn.

The statement that RL does not require a predefined set of strategies is mentioned to highlight the differences between this approach and other existing methods that are used to model animal behaviour, such as replicator dynamics. Having said that, we understand that the meaning was unclear. As mentioned in the response to Reviewer 1’s comment, we have changed the wording to instead highlight that through RL we do not need to constrain the strategy space from the outset. Replicator dynamics requires that the set of strategies to be analysed are defined at the outset – this set will be a subset of the entire strategy space. Then, typically, the population consists of sub-populations of agents following each chosen strategy proportionally to their fitness. Instead, RL does not have this constraint. The RL agents are able to learn any strategy in the entire strategy space, which is why we mention that RL does not require a predefined set of strategies (so there is no constraint on the strategy space). This is precisely one of the key advantages of using an RL approach like Q-learning: agents are able to explore and find optimal or near-optimal policies rather than being instantiated with a set strategy, as in replicator dynamics. The goal of the two approaches is different and can be complementary: replicator dynamics allows us to see the relative success and evolutionary stability of given strategies at population level, whereas reinforcement learning looks at individual agents and studies the optimal policy that they learn through repeated interactions. In the latter, agents can dynamically update their strategy in response to the environment, as animals are able to adapt to shifting conditions. Replicator dynamics is a useful method to understand the behaviour of populations over time, whereas RL more closely mimics individual learning: for instance, in this paper we assimilate the equilibrium obtained through RL to proactive prosociality, which is observed in animal behaviour.

In order to clarify this point in the manuscript, we have added to the manuscript (highlighted in the tracked changes document). Some of the additions include:

Lines 52-55:

“Reinforcement learning involves a defined action space, which are the possible actions that each agent may take, and a set of rewards that guide the learning process, but no predefined set of strategies. Using this approach, we are able to observe the strategies that emerge through interactions.”

Lines 101-108:

“The use of reinforcement learning, more specifically Q-learning in this case, only requires a defined action space, environment, and a set of learning parameters. By not requiring the strategy space to be defined from the outset, we can observe the strategies that emerge through repeated interactions. Agents initially explore randomly between the set of actions available to them, and update the expected reward of a given action. In this way, reinforcement learning allows the agents to arrive at the optimal or near-optimal strategy.”

3. ***Insufficient justification for use of RL****. The paper aims to show that MARL (multi-agent reinforcement learning) can be used to gain insight into “the underlying mechanics of learning over generations”, citing two key advantages of MARL as interpretability and the “predefined strategies” point. I believe that the significance of these advantages is overstated, that MARL makes more sense from an engineering perspective, and therefore that other strategy adaption mechanics should be explored.*

*In individual learning models, as this paper presents, RL allows agents to learn through trial and error and individual rewards. As an engineering tool, RL allows engineers to design agents that maximise long term rewards through exploration of the strategy space. I don’t believe this is a particularly realistic way to model strategic adaption in an evolutionary biology sense, where populations of agents are present and may be large (effectively infinite as in replicator-mutator dynamics) or small (e.g. agent-based models with weak selection). As is stated in the paper, it is true that in these models agents’ strategies are “predetermined” and change from one to another in a discontinuous way, but this argument also holds true for Q-learning: as soon as the Q-value for one action is larger than another then this action is chosen w.p. 1 − ϵ. Additionally, while Q-values are directly interpretable as the quality of a certain action in a certain state, the average utility of an individual playing strategy S can also be interpreted as such in a model with “predetermined” strategies. Finally, when using a technique such as adaptive dynamics, the probabilities of playing a certain strategy in a mixed strategy set can be updated in a continuous way such that there is no discontinuous switch between strategies. As such, I believe that the use of MARL is currently not sufficiently justified such that the entire paper centres on its use due to its comparative lack of biological realism, and lack of clear advantages over other methods in interpretability and flexibility.*

We value the discussion on the suitability of MARL for this case and appreciate the debate regarding its advantages over other methods. With respect to the point that MARL makes more sense from an engineering perspective and has shortcomings in its application to biology, we would like to point to existing literature that highlights the value of using reinforcement learning in biological settings (Frankenhuis, Panchanathan and Barto, 2019; Yamaguchi et al., 2018; Enquist, Lind and Ghirlanda, 2016; Whalen Cownden and Laland, 2016; Dridi and Lehmann, 2015a; 2015b). In a paper published in *PLOS Computational Biology*, Yamaguchi et al. (2018) apply inverse reinforcement learning (IRL) to study the behavioural strategies of animals. The following excerpt from the article discusses the suitability of reinforcement learning (RL) for their application:

*“RL is a mathematical paradigm that represents how animals adapt their behavior to maximize cumulative rewards via trial and error (Sutton and Barto, 1998) ... A previous study indicated that dopamine activity reflects the reward prediction error (Schulz, Dayan and Montague, 1997), similar to temporal difference learning in RL (Montague, Dayan and Sejnowski, 1996), suggesting that RL-based regulation underlies animal’s behavioral learning. Even in the simple neural circuits of Caenorhabditis elegans (C. elegans), dopamine-dependent activity, involved in explorative behavior, is reminiscent of RL (Calhoun et al., 2015). Thus some behavioral strategies are likely associated with the reward system.”*

Similarly, Frankenhuis, Panchanathan and Barto (2019) discuss how much behavioural ecology could benefit from drawing on reinforcement learning methods. They state that “reinforcement learning methods are well-suited to studying how biological mechanisms solve developmental and learning problems. For instance, we can use them to study simple rules that perform well in complex environments.”

As these papers suggest, RL has been shown to be a valuable tool to study (single) animal behaviour. In this research, we add to this body of scholarly work by looking at a multi-agent case, where there are two learning agents interacting. This multi-agent perspective allows us to explore the co-evolution of behaviour, looking at feedback loops in the agents’ behaviour. Although limited compared to single-agent RL scenarios, MARL has been used to study animal behaviour in areas including predatory-prey dynamics (Tsutsui, Takeda and Fujii, 2023; Yamada, Shawe-Taylor and Fountas, 2020) and collective/swarm movement (Hahn et al., 2019; Durve, Peruani and Celani, 2020). The reviewer points out that RL is perhaps less relevant as these interactions occur as part of a population. We concur that by modelling animal behaviour we are considering behaviour that will generally happen within a population that is not included in the model. Having said that, the individual interactions within this population are often dyadic in nature, therefore using the modelling approach presented here remains relevant.

We would also like to more closely address the following statement from the reviewer: “*As is stated in the paper, it is true that in these models agents’ strategies are “predetermined” and change from one to another in a discontinuous way, but this argument also holds true for Q-learning: as soon as the Q-value for one action is larger than another then this action is chosen w.p. 1 − ϵ.*” The reviewer is correct in stating that through Q-learning, the Q-values often converge after some time and, for a given state, a certain action will generally be taken. This is precisely one of the aspects that reinforcement learning, and within this Q-learning, is useful for. In fact, RL is used to find the optimal strategy, so this convergence is what we want to find out. The convergence is necessary to reach this objective. This point was not sufficiently clear, since it appears that the reviewer assimilates this to predetermined strategies in other approaches like replicator dynamics. The difference is that in this case they are not predetermined, so the convergence allows us to observe what appears to be the optimal strategy. With regards to the reviewer’s comment that after a certain point an action will always be chosen, the Q-values correspond to action-state combinations, which means that once the values converge, a certain action will only consistently be chosen when in that state. Also, reinforcement learning is dynamic and adaptative to environmental changes, meaning that even if the values have converged, a modification in the environment can result in a change in strategy as the Q-values are updated.

4. ***Modelling decisions are at odds with readers****. Many modelling decisions in the paper do not have a strong enough justification that they are biological plausible. A central part of PLOS Computational Biology is in providing insight into the mechanics of biological systems so any abstractions or deviations from biological reality should come with a strong justification in order for the results of the study to remain relevant.*

*For example, while Q-learning at an individual level has been used to model biological phenomena (see Neftci and Averbeck [2019]), the evolutionary perspective on utility taken by inclusive fitness happens at the level of genes and therefore at a generational timescale. Therefore, the biological plausibility of combining the individual learning of RL agents and the generational adaption of inclusive fitness must be justified.*

*Another example is found in the choice of a learning rate that is fixed for 50 rounds and then decays over time. While this may lead to learning outcomes that maximise long-term utility, there is no evidence given that this way of modelling agents using Q-learning leads to realistic dynamics either in terms of what is learned or the learning trajectories themselves. When one of the key claims of the paper is offering insight into a biological system through understanding its mechanics and the interpretability of the method, the ability of this method to accurately model such a system is important.*

With regards to the comment that the use of reinforcement learning, and more specifically Q-learning, may not be biologically plausible, we would like to point to a couple of *PLOS Computational Biology* papers that discuss how reinforcement learning algorithms relate to the way that learning happens in animals and so can be used to model learning in animals. Frémaux, Sprekeler and Gerstner (2013) discuss the presence of reward-based learning in animals, and state that reinforcement learning precisely provides a framework to study this type of learning. Blackwell, and Doya (2023) refer to Q-learning specifically as a model that can be used to explain learning behaviours. These papers provide evidence for the biological validity of this approach and for its relevance to *PLOS Computational Biology*. We agree with the reviewer that not all the parameters used in Q-learning and reinforcement learning can be directly mapped onto the real learning mechanisms within animal behaviour, however this approach is still valuable as a way of studying learning dynamics.

Inclusive fitness is indeed generally used to study gene-level evolution. However, Maynard Smith’s original paper formulating the Sir Philip Sidney game (Maynard Smith, 1991) set out to illustrate previous arguments about honest signalling in animal communication such as fights or sexual selection. His use of inclusive fitness is not at gene-level but instead looks at signalling and communication between animals. In modelling the Sir Philip Sidney game using reinforcement learning, we are maintaining the original formulation of the game, which includes the use of inclusive fitness rather than individual rewards (we have clarified that this was part of the original formulation in the text). Subsequent papers have similarly used inclusive fitness in this game to study animal behaviour. For instance, Johnstone and Grafen (1992) specifically use the example of vampire bats begging for blood when discussing the Sir Philip Sidney game, making it clear that they are using inclusive fitness and this game framework to model animal behaviour rather than gene-level dynamics.

Regarding the comment about the learning rate decay, we would like to point out that the learning rate remains constant throughout the simulation; it is the exploration rate that decays after the first 50 rounds. We believe that the mechanism in itself has a biological mapping, i.e., animals tend to explore less after they acquire sufficient information about their environments and interactions with others. Having said that, we agree with the reviewer that the choice of the exact number of rounds after which it decays is somewhat arbitrary and therefore less biologically plausible or interpretable. For this reason, we chose to run the simulation with varying exploration rates and initial lengths of exploration, in order to see whether this had a significant impact on the learning trajectories and strategies. These results are included in the Appendix. We note that there is no significant difference in learning with these variations: the same strategies are always learned the majority of the time, what changes is the proportion of runs that they are learned. With longer exploration lengths or less decay, there is stronger convergence to the optimal strategy.

Overall, previous literature has demonstrated that RL is a valuable tool for studying animal behaviour and offers a degree of biological interpretability. While there are some aspects, such as the initial exploration period which then decays, that are less able to be mapped onto the real mechanisms, this does not negate the utility of using reinforcement learning as a tool to study animal behaviour.

We would like to thank the reviewers again for their valuable comments. We believe that these changes have significantly strengthened the manuscript while preserving its core insights, and we are grateful to the reviewers for helping us to improve this work and add necessary clarifications.

Sincerely,

The authors

References

Blackwell, K.T. and Doya, K. (2023) Enhancing reinforcement learning models by including direct and indirect pathways improves performance on striatal dependent tasks, *PLOS Computational Biology*, 19(8).

Calhoun, A.J. *et al.* (2015) Neural mechanisms for evaluating environmental variability in Caenorhabditis elegans, *Neuron*, 86(2), pp. 428–441.

Dridi, S. and Lehmann, L. (2015a) A model for the evolution of reinforcement learning in fluctuating games, *Animal Behaviour*, 104, pp. 87–114.

Dridi, S. and Lehmann, L. (2015b) Environmental complexity favors the evolution of learning, *Behavioral Ecology*, 27(3), pp. 842–850.

Durve, M., Peruani, F. and Celani, A. (2020) Learning to flock through reinforcement, *Physical Review E*, 102(1).

Enquist, M., Lind, J. and Ghirlanda, S. (2016) The power of associative learning and the ontogeny of optimal behaviour, *Royal Society Open Science*, 3(11), p. 160734.

Frankenhuis, W. E., Panchanathan, K., and Barto, A. G. (2019) Enriching behavioral ecology with reinforcement learning methods. Behavioural Processes, 161, pp.94–100.

Frémaux, N., Sprekeler, H. and Gerstner, W. (2013) Reinforcement learning using a continuous time actor-critic framework with spiking neurons, *PLoS Computational Biology*, 9(4).

Hahn, C., Phan, T., Gabor, T., Belzner, L. and Linnhoff-Popien, C. (2019) Emergent Escape-based Flocking behavior using Multi-Agent Reinforcement Learning. Proceedings of the The 2019 Conference on Artificial Life (ALIFE 2019). pp. 598-605.

Huttegger, S.M., Zollman, K.J.S. (2010) Dynamic stability and basins of attraction in the

Sir Philip Sidney game. *Proceedings of the Royal Society B: Biological Sciences*, 277(1689), pp.1915–1922.

Johnstone, R.A. and Grafen, A. (1992) The continuous Sir Philip Sidney game: A simple

model of biological signalling. *Journal of Theoretical Biology*, 156(2), pp.215–234.

Maynard Smith, J. (1991) Honest signalling: the Philip Sidney game. *Animal Behaviour*, 42, pp.1034–1035.

Montague, P., Dayan, P. and Sejnowski, T. (1996) A framework for mesencephalic dopamine systems based on predictive Hebbian Learning, *The Journal of Neuroscience*, 16(5), pp. 1936–1947.

Schultz, W., Dayan, P. and Montague, P.R. (1997) A neural substrate of prediction and reward, *Science*, 275(5306), pp. 1593–1599.

Sutton, R.S. and Barto, A.G. (1998) *Reinforcement Learning: An Introduction*. Cambridge, MA: The MIT Press.

Tsutsui, K., Takeda, K., Fujii, K. (2023). *Emergence of Collaborative Hunting via Multi-Agent Deep Reinforcement Learning*. In: Rousseau, JJ., Kapralos, B. (eds) Pattern Recognition, Computer Vision, and Image Processing. ICPR 2022 International Workshops and Challenges. Lecture Notes in Computer Science, vol 13643. Springer, Cham.

Whalen, A., Cownden, D. and Laland, K. (2015) The learning of action sequences through social transmission, *Animal Cognition*, 18(5), pp. 1093–1103.

Yamada, J., Shawe-Taylor, J. and Fountas, Z. (2020*). Evolution of a Complex Predator-Prey Ecosystem on Large-scale Multi-Agent Deep Reinforcement Learning*. *arXiv preprint*: 2002.03267.

Yamaguchi, S. *et al.* (2018) Identification of animal behavioral strategies by inverse reinforcement learning, *PLOS Computational Biology*, 14(5).
