# Supplementary material for: Maynard Smith revisited: A multi-agent reinforcement learning approach to the coevolution of signalling behaviour
Source: PLoS Comput Biol. 2025 Aug 26;21(8):e1013302. doi: 10.1371/journal.pcbi.1013302 (PMC12440204; doi:10.1371/journal.pcbi.1013302)
Supplement: Attachment — Submitted filename: Response_to_Reviewers_auresp_2.docx [file pcbi.1013302.s008.docx]

**Rebuttal Letter**

The authors would like to thank the editor and reviewers again for taking the time to go through the updated version of the manuscript, and for providing a new round of comments. We greatly appreciate the opportunity to resubmit our revised paper for your renewed consideration. Below we respond to the editor’s and reviewers’ comments and point to the corresponding changes we made in the paper. We have inserted in this letter some of the text that has been added been to the manuscript, along with line numbers. We have also submitted the new version of the manuscript with all changes highlighted.

## **Editor**

1. *Update and document the GitHub code so it is up-to-date and runs out-of-the-box.*

We have now updated the code on the GitHub repository. The current code is similar to what was already in the repository; the logic and implementation of the experimental simulation remains the same, the changes are mainly improving code efficiency and readability.

2. *Provide a detailed, explicit account of the Q-learning implementation, including state definition, discount factor, strategy-space limits -- and demonstrate results for γ = 0 -- to rule out potential artifacts.*

We have included a more detailed explanation in the response to Reviewer 2’s second comment. There was a lack of clarity regarding the state that is being used to update the Q-value. We have clarified both in this letter and in the text that the agent is not predicting what action their opponent will take. Instead, the agent only updates the Q-value for the previous action once they know what action their opponent will take next (so the state they will be in when taking the next action).

With regards to the discount factor, the reviewer’s question was in relation to the use of the state in the Q-value formula. Having a discount factor of 0 (*γ = 0*) results in the formula:

$$Q_{t}(s, a) = {(1-\alpha)(Q}_{t-1}(s, a) + \alpha(R_{t}(s, a))$$

Using this formula, the Q-values only depend on the value of the reward $R$ and the learning rate $\alpha$. If the opponent always plays the same action (e.g. *Always Give*), the Q-value will quickly converge to the player’s reward for the action taken when the opponent chooses to *Give* – the speed at which this happens will depend on the learning rate $\alpha$. Otherwise, depending on the distribution of actions, the Q-value will converge to the expected reward in a one-shot game given the opponent’s action distribution. There is no consideration of future rewards, so each round essentially becomes a one-shot game.

We have also clarified constraints on the strategy space in the response to Reviewer 2’s first comment and in the text. Although the memory length does play a role in the strategies that are learned by the agents, due to their having increased observability, this does not constrain the strategies that the agent could learn.

## **Reviewer 1**

1. *The authors have addressed all my comments very well. I really appreciate their great efforts to make everything much clearer. I believe this is a very good contribution to the theoretical literature of evolution of prosocial and collective behaviours. I am happy to recommend the paper publication in its present form.*

We are grateful to Reviewer 1 for their positive comments and their constructive suggestions in the previous round. We believe that adding the clarifications previously suggested around MARL strategies and potential emergent behaviour had significantly strengthened the paper.

## **Reviewer 2**

1. *I am still not happy with the "RL doesn't constrain the strategies that can be learned" argument. … As soon as the state space is determined, the set of learnable strategies is determined. I'm not just being pedantic about choice of words here. Throughout the introduction of the paper (lines 37 to 114), the authors reiterate that, while previous work studying the PS game exists, in this paper MARL is used so you can study the emergence of decision-making strategies without specifying which ones are learnable. I just don't think this is true. Once you specify the agents' memory length, both agents are learning policies from a now specified strategy space. Please explain whether I am misunderstanding something or justify your use of this statement beyond the explanations given.*

The reviewer’s comment follows on from a discussion in the last round where we mentioned that reinforcement learning (RL) does not require a predefined set of strategies. The reviewer is correct in that there are constraints on the strategy space imposed by the agent’s architecture. We want to distinguish between constraints imposed by the design of the agent (which are present in RL), and constraints imposed by hand-coding which subset of strategies are available to the agent (as in replicator dynamics). There is a crucial difference in the objective: whereas replicator dynamics aims to study which strategies are selected from a fixed set defined by the researcher, RL allows us instead to investigate which strategies emerge from a (much larger) strategy space that the agent is able to explore given its architecture. The reason this distinction is relevant is that RL allows for the discovery of strategies that may not have been anticipated or explicitly programmed. Therefore, while the strategy space is indeed bounded by the choice of hyperparameters and agent architecture, we do not constrain it beyond this (i.e., we do not constrain it beyond the set of learnable strategies). The central distinction is between explicitly programming specific strategies, and defining the constraints within which strategies can emerge, then observing which ones actually develop through learning.

As the reviewer points out, certain factors do impact what strategies are learned. The main factor is the state space, which dictates what strategies are learned by the agents through exploration and exploitation. For instance, if the agent only has access to its own past actions and not the opponent’s, it will learn a different set of strategies than if it is able to observe both their own and the opponent’s five previous actions. To address this, we can vary the memory that the agent has. The memory size defines the state space and so affects the strategies that are learned. We do precisely this in the Appendix, looking at memory lengths of 1, 2, 3 and 4. As we note in the article, the strategy that is learned most often does not change with variations in memory size, but with increasing memory length there is greater variation in the distribution of strategies learned.

We are grateful to the reviewer for pointing out the choice of words, as we agree that the distinction is nuanced. We have made changes in the text to clarify this point. We include here two examples, where we have added the text in bold (lines 90-91 and 111-112):

*“In using MARL, there is no requirement to constrain the set of possible strategies that agents may learn* ***beyond architectural constraints imposed by the agent’s design, primarily memory length.****”*

*“In contrast to other methods, reinforcement learning does not require the strategy space to be constrained* ***beyond the set of learnable strategies defined by the agent's architecture.****”*

2. *I don't understand how the agents can effectively use the discount factor as I don't see how they can predict the next state given their current state and action. … please explain what the next state is. In line 293 you specify that the state in the state-action pair is "made up of both players' actions in the previous round", hence in equation (2) Q(s', a') refers to s' which is the next state, made up of both players' actions in the current round. How can the signaller know their interaction partner's action in the current round? If they don't know what the next action is e.g. if s' is just s (which is the case in the version of the code in the linked Github repository), then this term is just noise and it makes sense that "for each value of learning rate, a lower discount rate leads to the optimal strategy being learned a higher proportion of the time." (line 363). My worry is that the cyclical/alternating strategies are not an emergent property but just a byproduct of the agents thinking they can predict the future i.e. what action their opponent will take, and the various sources of noise in the reward signal. What happens when the discount factor is zero? Are cyclical strategies learned?*

To clearly address this comment, we have inserted Equation (2) from that paper, as this is the one being referred to:

$$Q_{t}(s, a) = Q_{t-1}(s, a) + \alpha(R_{t}(s, a) + \gamma\max_{a'} Q(s', a') - Q_{t-1}(s, a))$$

We would like to note that this is the standard Q-learning formula, first introduced by Watkins and Dayan (1992, p.280) in a slightly different form, and then used extensively in the form we have presented (see for example Jang et al. (2019) for this form of the equation (p.133656) and an overview of applications).

The reviewer is referring in particular to the term that is multiplied by the discount factor:

$$\max_{a'} Q(s', a')$$

There seems to be some confusion around when the Q-value is updated. The Q-value for the previous action-state pair $Q_{t}(s, a)$ is updated once both players have already played the next move, therefore the player knows what state they will be in when they take the next action, so knows $s'$. Given this state, the player multiplies the discount factor by the available action with the highest Q-value (so highest expected value). In a sense, this can be conceptualised as the ‘target Q-value’ that represents the situation that the player would like to end up with in the next turn.

In the code shared in the GitHub repository, the expression that corresponds to the above term is:

np.argmax(self.player.q_table[self.player.state_dict[state], action])])

Here we are also taking the maximum Q-value for this state (note the use of $max$ in both cases). The agent is not predicting their opponent’s next move; instead, they only update the previous Q-value once they know what state they will be in when taking their next action. $s$ refers to the state that an agent is in when they are taking an action, therefore when at the time of updating Q-value $Q_{t}$ they are not yet in state $s'$. When it comes to taking the next action $a'$, the agent will then be in state $s'$. We understand that this was not clear enough in the text or in our previous explanation and have added the below text to the manuscript (lines 308-313):

*“The agent updates the Q-value* $Q_{t}$ *for the action they just played once they know the reward they received, meaning that they know the action just played by the opponent and so the state they will be in when taking the next action. The term that is multiplied by the discount factor* $\gamma$ *represents the value of the action with maximum expected reward given the state they will be in when taking this next action.”*

With regards to what happens with the discount factor is 0, as explained in response to the Editor’s comment, this results in the Q-values converging to the expected reward for that action in a one-shot game given the opponent’s action distribution. This can be seen when examining the equation if we set $\gamma=0$, which becomes:

$$Q_{t}(s, a) = {(1-\alpha)(Q}_{t-1}(s, a) + \alpha(R_{t}(s, a))$$

As we can see, the Q-value now only depends on the learning rate and the rewards received by the agent for the action taken. Whether this results in cyclical strategies would depend on the strategy of the opponent.

We would like to thank the reviewers again for their valuable comments. We hope that points that were not fully clarified in the previous round of revision have now been explained to a satisfactory level. We are grateful for this second round of reviews, as we agree that these points need to be as clear as possible in the manuscript. We are grateful to the reviewers for helping us to improve this work and add necessary clarifications.

Sincerely,

The Authors

References

Watkins, C.J.C.H. and Dayan, P. *Q*-learning. *Machine Learning,* 8, pp.279–292 (1992).

Jang, B., Kim, M., Harerimana, G. and Kim, J.W., Q-Learning Algorithms: A Comprehensive Classification and Applications, *IEEE Access*, 7, pp.133653-133667 (2019).
